# HibeRNAtion: HIV-1 RNA Metabolism and Viral Latency

**DOI:** 10.3389/fcimb.2022.855092

**Published:** 2022-06-14

**Authors:** Raquel Crespo, Shringar Rao, Tokameh Mahmoudi

**Affiliations:** ^1^ Department of Biochemistry, Erasmus University Medical Center, Rotterdam, Netherlands; ^2^ Department of Pathology, Erasmus University Medical Center, Rotterdam, Netherlands; ^3^ Department of Urology, Erasmus University Medical Center, Rotterdam, Netherlands

**Keywords:** HIV-1, rna processing, viral latency, post-transcriptional regulation, HIV-1 rna

## Abstract

HIV-1 infection remains non-curative due to the latent reservoir, primarily a small pool of resting memory CD4+ T cells bearing replication-competent provirus. Pharmacological reversal of HIV-1 latency followed by intrinsic or extrinsic cell killing has been proposed as a promising strategy to target and eliminate HIV-1 viral reservoirs. Latency reversing agents have been extensively studied for their role in reactivating HIV-1 transcription *in vivo*, although no permanent reduction of the viral reservoir has been observed thus far. This is partly due to the complex nature of latency, which involves strict intrinsic regulation at multiple levels at transcription and RNA processing. Still, the molecular mechanisms that control HIV-1 latency establishment and maintenance have been almost exclusively studied in the context of chromatin remodeling, transcription initiation and elongation and most known LRAs target LTR-driven transcription by manipulating these. RNA metabolism is a largely understudies but critical mechanistic step in HIV-1 gene expression and latency. In this review we provide an update on current knowledge on the role of RNA processing mechanisms in viral gene expression and latency and speculate on the possible manipulation of these pathways as a therapeutic target for future cure studies.

## 1 Introduction

With more than 37 million people living with, Human Immunodeficiency type-1 (HIV-1) infection remains a prevalent global health burden (Organization WHO, 2022). Although the introduction of antiretroviral therapy (ART) in the 90s transformed a once lethal disease into a chronic condition, widespread inequalities in treatment accessibilities, the development of resistance and the economic burden of lifelong ART warrant the need for curative therapies. The main obstacle to the development of an HIV-1 cure is the establishment of a particularly complex HIV-1 viral reservoir in the absence of viral replication under effective ART, that is responsible for viral rebound upon treatment cessation (Finzi et al., 1997; Chun et al., 2015). Viral reservoirs are established quickly in the acute phase of infection and are formed by a heterogeneous population of long-lived replication-competent latently infected cells (Kreider and Bar, 2022; Siliciano and Siliciano, 2022). Latency is induced and maintained at multiple levels in the HIV-1 replication cycle such as inhibition of transcription initiation and elongation, inhibition of RNA processing and translation of vRNA into viral proteins and is hence defined as the reversivel absence of active viral production by HIV-1 infected cells (Telwatte et al., 2019; Dufour et al., 2020; Siliciano and Siliciano, 2022). Several strategies have been proposed to target, reduce and/or eliminate latently-infected cells of the viral reservoir (Deeks et al., 2021). A widely-investigated approach aims at pharmacological reactivation or “shocking” of the HIV-1 provirus in order to produce viral RNA or proteins that can trigger intrinsic cell death or immune-mediated cytotoxic killing (Deeks, 2012). Clinical studies have demonstrated that although the “shock” strategies have been shown to be effective *in vivo* to reactivate HIV-1 transcription, there has been negligible to no reduction in the size of the HIV-1 reservoir (Kim et al., 2018). It is estimated that current latency-reversing agents (LRAs) are able to reactivate only 5% of latently- infected cells and that only 2-10% of cells that produce HIV-1 viral RNA (vRNA) also make viral proteins (Grau-Exposito et al., 2019). This is due to the complex nature of latency, which involves strict intrinsic regulation at multiple transcriptional and post-transcriptional levels. The transcriptional regulation of HIV-1 latency is well characterized, and most known LRAs target LTR (long terminal repeats)-driven transcription by countering chromatin mediated repression, or facilitating transcription initiation or elongation (Ait-Ammar et al., 2019). However, the co and post transcriptional events that regulate HIV-1 vRNA metabolism have been relatively understudied. In this review, we outline the various steps of HIV-1 vRNA processing and describe how post-transcriptional control of HIV-1 and its misregulation can contribute to latency. Finally, we speculate about possible therapeutic approaches in targeting post-transcriptional steps of vRNA regulation and their potential implications in HIV-1 cure research.

## 2 What’s RNA Got to Do With it? The Contribution of RNA-Binding Proteins to HIV-1 Latency

Transcription of the HIV-1 genome is controlled by a promoter located in the 5’ long terminal repeat (LTR) of the provirus (Verdin, 1991; Verdin and Van Lint, 1995; Ne et al., 2018). The transcriptional activation state of the HIV-1 promoter is regulated by the availability of host transcription initiation and elongation factors, chromatin landscape of the LTR and the action of viral proteins (Van Lint et al., 2013; De Crignis and Mahmoudi, 2017). There is a certain degree of stochasticity in HIV-1 transcriptional activation, resulting from transitional activation or repression of transcription due to cell cycle dependent fluctuations and availability of cell host co-factors (Weinberger et al., 2005; Ho et al., 2013).

While the different steps of gene expression entailing transcription, 5’ capping, splicing, and cleavage/polyadenylation are usually investigated independently of one another, these processes occur simultaneously, are closely coupled and localized to the same regions in the nucleus (Beyer and Osheim, 1988; Rasmussen and Lis, 1993; Bauren et al., 1998). Host cell factors involved in these processes are recruited co-transcriptionally and nascent RNA is processed instantly after synthesis by the RNA Polymerase II (Moore and Proudfoot, 2009; Nojima et al., 2015).

**Figure 1:**
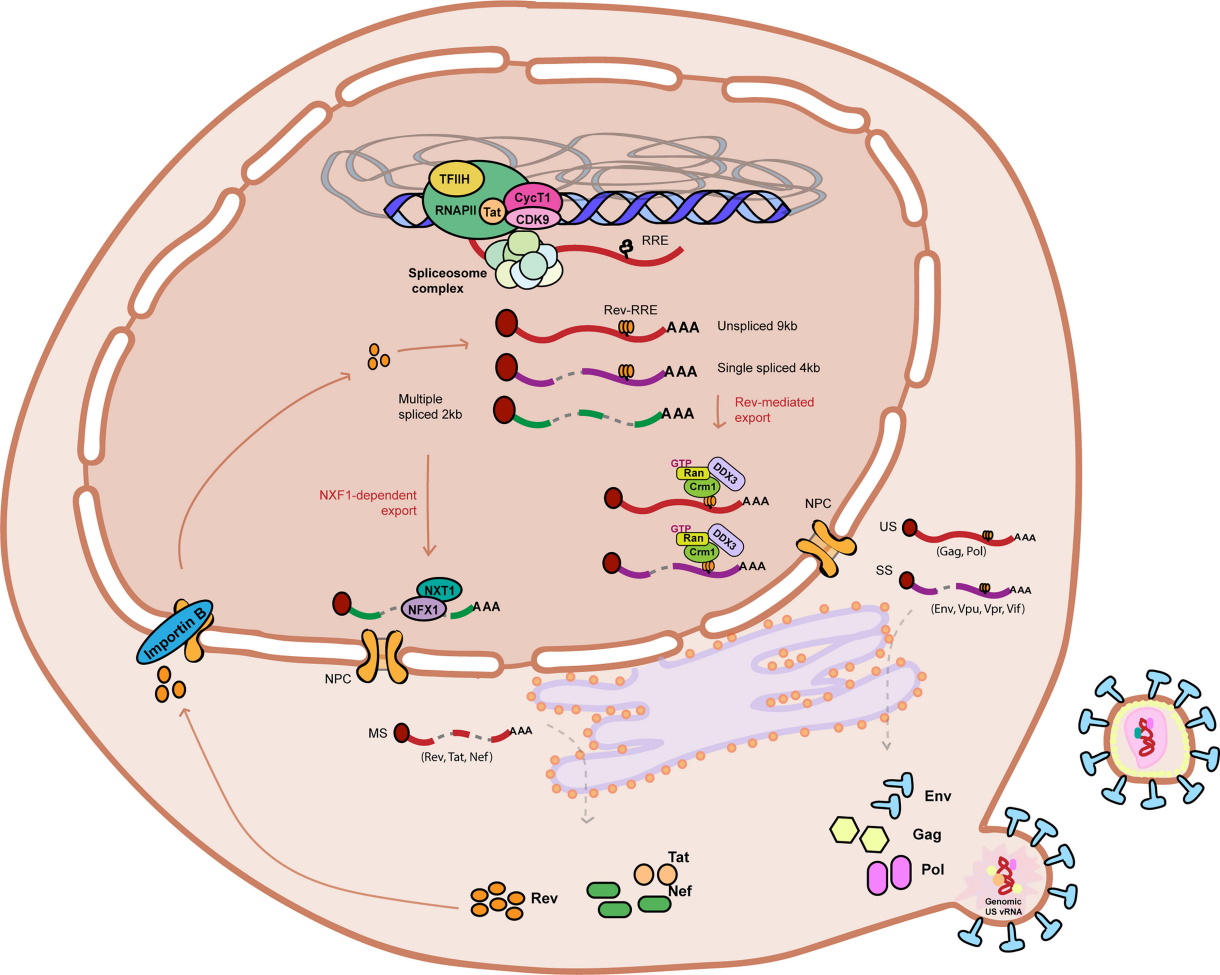
Graphical representation of HIV-1 RNA metabolism and gene expression. HIV-1 RNA is actively transcribed from the HIV-1 promoter by the RNA Polymerase II in complex with Positive Transcription Elongation Factor B (PTEF-b) complex and viral protein Tat amongst other co-factors. Nascent viral RNA is processed co-transcriptionally upon 5’ capping (indicated by a red coloured circle at the beginning of the vRNA), recruitment of the host cell spliceosome machinery, and polyadenylation. The Unspliced (US) 9kb vRNA is subjected to alternative splicing and results in two other vRNA splicing variants: Single spliced (SS) 4kb vRNA and Multiple spliced (MS) 2kb vRNA. MS vRNA transcripts are exported *via* NXF1/NXT1-mediated export and translated in the cytoplasm to produce HIV-1 proteins Tat, Nef and Rev. Nucleocytoplasmic export of US and SS vRNA transcripts is dependent on Rev (imported to the nucleus by Importing B) binding to the secondary RNA structure RRE. Multiple Rev molecules multimerize and bind RRE-containing US and SS vRNA transcripts and recruits host cell protein CRM-1 to form an RNP containing other host cell proteins such as Ran-GTP and DDX3, promoting its export into the cytoplasm. US and SS vRNA transcripts are translated in the cytoplasm to produce, respectively, Gag and Pol polyproteins and envelope protein Env and accessory proteins Vpu, Vpr and Vif. Viral assembly, budding and maturation are the last steps of the HIV-1 life cycle. Unspliced 9kb viral RNA is represented as a red curved line. Single spliced 4kb viral RNA is represented as a partially cut purple curved line. Multiple spliced 2kb viral RNA is represented as a partially cut green curved line. RRE stands for Rev response element. NPC stands for nuclear pore complex.

The HIV-1 vRNAs, similar to host cell mRNAs, need to undergo various steps of co and post-transcriptional RNA processing to ensure viral gene expression and the production of infectious viral particles. Viral RNA processing starts co-transcriptionally with the recruitment of various RNA-binding proteins during transcription (**Figure 1**). The mRNAs are capped and polyadenylated and subjected to epitranscriptomic modifications such as m6A or m5C methylation (Meyer and Jaffrey, 2014; Kong et al., 2019). The 9kb HIV-1 unspliced vRNA (HIV-1 US vRNA), that also serves as genomic viral RNA, is subjected to alternative splicing to generate either the 4kb singly spliced vRNAs (HIV-1 SS vRNA) or 2kb multiply spliced vRNAs (HIV-1 MS vRNA) transcripts (Purcell and Martin, 1993; Stoltzfus, 2009). The formation of distinct ribonucleoprotein complexes (RNPs) are necessary for the trafficking of the vRNAs and to mediate their nucleocytoplasmic export, where they can be translated to generate viral proteins (Cochrane et al., 2006). Aberrant splicing, mislocalization of the vRNAs, nuclear or cytoplasmic degradation of misprocessed vRNAs or inefficient translation of the vRNAs can all result in reduced viral gene expression, thereby enforcing viral latency. All of these processes involved in RNA metabolism are mediated by numerous host RNA binding proteins (RBPs) (Cochrane et al., 2006). Characterization of the roles of RBPs in regulation of HIV-1 gene expression and viral latency will therefore reveal potential new targets for pharmacological manipulation in context of HIV cure strategies.

Multiple studies have characterized the HIV-1 vRNA interactome and the contributions of these proteins to viral gene expression (Kula et al., 2011; Knoener et al., 2017; Knoener et al., 2021). Several medium/high-throughput unbiased omics approaches, have aimed at characterizing RNA processing proteins and complexes specifically involved in regulation of HIV-1 latency. Transcriptomic studies in *in vitro* infected primary cell models of latency and primary CD4+ T cells obtained from HIV-1 infected donors have shown that post-transcriptional blocks are seemingly controlled by multiple host cell proteins involved in RNA metabolism and processing, proteins that belong to the spliceosome complex, and long non-coding RNAs differentially expressed in unstimulated and stimulated conditions (Golumbeanu et al., 2018; Moron-Lopez et al., 2020). Moron-Lopez et al. described in their study more than 5000 differentially expressed (DE) genes in unstimulated and stimulated cells obtained from HIV-1 infected donors, and a total of 234 DE genes shared between multiple primary cell models of latency and cells obtained from HIV-1 donors, indicating the differences between these models in regulation of HIV-1 latency and latency reactivation (Moron-Lopez et al., 2020). Amongst those, there was enrichment of multiple genes involved in RNA metabolism, such as members of the spliceosome complex and Serine and Argining-rich (SR) proteins, poly-A binding proteins such as PolyA Polymerase Alpha (PAPOLA), or mRNA decay pathway members such as Mago Homolog, Exon Junction Complex Subunit (MAGOH). In another study, Golumbeanu et al., using single-cell RNA sequencing, observed more than 130 differentially expressed genes in latent versus histone deacetylase inhibitor SAHA and TCR-triggering activated primary CD4 T cells *in vitro* infected and in cells obtained from two HIV-1 infected donors (Golumbeanu et al., 2018). These genes mostly belonged to ribosomal components and other members of RNA processing pathways such as Dead-box helicase 5 (DDX5), an RNA helicase involved in multiple levels of mRNA processing, and SRSF5, a component of the pre-mRNA spliceosome complex, and were already reported to have a role in HIV-1 RNA metabolism and gene expression (Manley and Krainer, 2010; Zhou et al., 2013; Sithole et al., 2020). Liu et al. have also shown that latently infected cells that induce viral RNA after latency reactivation are enriched for RNA binding proteins and members of the nonsense-mediated RNA decay such as UPF2 (Liu et al., 2020).

In our own work, we have recently shown in two independent unbiased omics studies that proteins involved in RNA metabolism are prominent regulators of HIV-1 latency (Röling et al., 2021). Using a haploid genetic screen we identified close to 70 host genes that are putatively involved in promoting or maintaining HIV-1 latency. Among those, we found that 10% of the proteins identified in our haploid screen are involved in host cell RNA processing and metabolism, such as DDX46 (also known as PRP5), involved in spliceosome assembly and RNA-protein interactions (Cordin and Beggs, 2013) or EIF2B5, involved in translation initiation (König et al., 2008). In another study, we performed locus-specific chromatin pulldown of the HIV-1 5´LTR latent versus active promoter coupled with mass spectrometry in order to identify differentially abundant putative repressors or activators of latency (in press (Ne et al., 2022)). We found that 50% of the putative repressor proteins bound to the latent promoter were proteins involved in RNA processing and metabolism, including members of the TREX1 and TREX2 complexes (DDX39A and PCID2) (Heath et al., 2016; Stewart, 2019), and RAMAC/FAM103A1, required for mRNA cap methylation (Gonatopoulos-Pournatzis et al., 2011). This work further enforces the notion that RBPs that we found present on the latent HIV-1 promoter are recruited co-transcriptionally and can contribute to viral latency.

## 3 Co and Post-Transcriptional Regulation of HIV-1 Gene Expression and Implications in Viral Latency

The degree to which co and post-transcriptional regulation contributes to HIV-1 latency has become clearer recently from single cell studies in cell line and primary cell latency models and CD4+T cells from ART-suppressed people living with HIV-1 (PLWH) (Yukl et al., 2018; Telwatte et al., 2019). Yukl and colleagues described that the relative contribution of transcriptional and post-transcriptional blocks to HIV-1 latency in CD4+ T cells obtained from ART suppressed individuals is mostly regulated by blocks in transcription completion, polyadenylation and RNA splicing (Yukl et al., 2018; Telwatte et al., 2019). These post-transcriptional blocks seem to be reversible upon TCR triggering, as determined by the significant increase in detection of spliced transcripts (MS vRNAs encoding Tat and Rev) and a decrease in initiated transcripts (e.g. short vRNAs) and US vRNA, thus confirming that blocks to splicing significantly repress HIV-1 post-transcriptionally. This is also the case for *in vitro* infected primary cell models of latency, in which blocks to splicing are a prominent obstacle preventing HIV-1 gene expression (Moron-Lopez et al., 2020). Importantly, RNA processing mechanisms, such as splicing, are also important in regulating HIV-1 latency in tissue-resident reservoirs, where the vast majority of latent HIV-1 resides (Telwatte et al., 2018). This significantly differs from cell line latency models, in which HIV-1 latency seems to be regulated by blocks in transcription initiation or elongation (Telwatte et al., 2019). Most of our current knowledge on the molecular mechanisms involved in HIV-1 latency, however, originates from studies that employ latent cell lines. This is due to technical feasibility of studies on cell line models, and lack of access to primary patient cells, and partly explains why co- and post-transcriptional mechanisms involved in latency have been historically understudied. The role of HIV-1 RNA biogenesis in HIV-1 latency is now being actively researched as technical advancements and studies on relevant model systems become available.

In the next sections, we describe the various steps involved in mRNA processing that have a characterized role in influencing viral gene expression, and we discuss their contributions to viral latency.

### 3.1 Splicing

The HIV-1 genome encodes viral proteins in a single unspliced polycistronic transcript of 9.2 kb (Purcell and Martin, 1993; Bohne et al., 2005; Stoltzfus, 2009). The HIV-1 US vRNA contains at least four 5’ splice donor sites and ten 3’ splice acceptor sites and the host cell spliceosome combines multiple splice donors and acceptors, that facilitates alternative splicing resulting in the production of over 100 HIV-1 vRNA transcripts of 4kb HIV-1 SS vRNA and 2 kb HIV-1 MS vRNA (Purcell and Martin, 1993; Ocwieja et al., 2012; Emery et al., 2017; Pasternak and Berkhout, 2021). HIV-1 US vRNA encodes for the structural precursor proteins Gag and Gag-Pol and serves as genomic RNA. The 4kb SS vRNA and 2kb MS vRNA encode for Env and accessory proteins (Vif, Vpu, Vpr), and Tat, Rev and Nef respectively (**Figure 1**). Early in the infection, HIV-1 vRNA processing depends fully on the host spliceosome machinery and only the HIV-1 MS vRNAs are exported to the cytoplasm and translated into the early proteins Tat and Rev, while the larger HIV-1 SS and US vRNAs accumulate in the nucleus (Stoltzfus, 2009; Karn and Stoltzfus, 2012). Rev protein then facilitates the cytoplasmic export of the larger HIV-1 SS and US vRNAs (See section 3.2) (Schwartz et al., 1992; Schwartz et al., 1992; Karn and Stoltzfus, 2012). The expression levels of Tat and Rev, which are encoded by the multiply spliced transcripts, determine a critical switch to a positive feedback loop of viral transcription, RNA export and processing, and protein production, which drive the production of viral particles during active infection. Tat binds the TAR RNA secondary structure in the nascent transcript at the 5’ LTR and recruits P-TEFb, leading to phosphorylation of the paused RNA Polymerase II and promotion of transcription elongation (Roy et al., 1990; Mancebo et al., 1997; Sedore et al., 2007). Absence of Tat protein below a critical threshold level dramatically reduces vRNA production and breaks the feedback loop necessary for sufficient transcriptional output necessary to maintain active viral production (Das et al., 2011; Kamori and Ueno, 2017). As Tat and Rev become available, a feedback loop of transcription activation and export of US and SS vRNA allows for efficient viral production.

Splicing of the HIV-1 US vRNA starts by recognition of sequences flanking the introns by the spliceosome. The US vRNA together with the proteins that constitute the spliceosome form an RNP that initiates and regulates splicing (Moore and Sharp, 1993). Regulation of HIV-1 vRNA alternative splicing is mediated by cis and trans-acting elements that allow for control of HIV-1 gene expression [reviewed in (Stoltzfus and Madsen, 2006; Caputi, 2011)]. Cis-acting splicing regulatory elements that control HIV-1 splicing function as exonic or intronic splicing enhancers (ESEs, ISEs) or silencers (ESSs, ISSs). The HIV-1 genome contains a total of 4 ESSs, 1 ISS, 1 ISE and 6 ESEs (Bohne et al., 2005; Stoltzfus, 2009; Karn and Stoltzfus, 2012). The most well-known family that act on exonic splicing enhancers (ESEs) are SR proteins that promote active splicing upon recognition of enhancer sequences by recruiting the splicing complex and have a role in mRNA export, stability and translation (Graveley, 2000; Caputi and Zahler, 2002; Huang and Steitz, 2005; Fukuhara et al., 2006; Stoltzfus, 2009; Jablonski and Caputi, 2009). Negative regulation of splicing depends largely on the activity of hnRNPs, proteins that act on exonic splicing silencers (ESSs) by preventing binding of the spliceosome complex (Caputi et al., 1999; Caputi and Zahler, 2002; Jablonski and Caputi, 2009; Sertznig et al., 2018). Tat and Rev have also been shown to promote or inhibit splicing of vRNAs by direct binding to HIV-1 US vRNA or by regulation of host splicing factors, although the exact contribution of Rev to alternative splicing is still unclear (Kjems et al., 1991; Kjems and Sharp, 1993; Stoltzfus, 2009; Jablonski et al., 2010; Mueller et al., 2018).

Trans-acting elements such as secondary structures present across the HIV-1 US vRNA also regulate HIV-1 splicing by hiding or exposing splicing sites to the splicing machinery (Saliou et al., 2009). Splicing donors and acceptors can be contained within RNA secondary structures that facilitate their auto-regulation. For example, splicing donor D1 is present in a stem loop RNA structure that negatively regulates its accessibility to the splicing machinery and impacts splicing efficiency (Abbink and Berkhout, 2008). As well, splicing acceptors A1 and A3 are known to be contained within a secondary RNA structure shown to be important for promoting HIV-1 US vRNA splicing (Ocwieja et al., 2012).

#### 3.1.1 Link to Viral Latency

Our understanding of the role of post-transcriptional regulation of HIV-1 splicing, and to what extent these mechanisms contribute to latency has evolved since the first reports demonstrating a temporal shift in the production of HIV-1 US and MS vRNA species (Pomerantz et al., 1990; Michael et al., 1991; Seshamma et al., 1992; Li et al., 1996). Presence of US vRNA, that encodes for structural proteins required for viral particle formation, was considered a sign of productive infection, and high US/MS vRNA ratios are present in later stages of the HIV-1 replication cycle. In the first cell line models of latency, such as U1 or ACH2, a much lower US/MS vRNA was present, indicative of a putative post-transcriptional block to HIV-1 viral production (Pomerantz et al., 1990; Seshamma et al., 1992). The first few hours upon activation with PMA or PHA, resulted in increased levels of MS vRNA, and a shift towards the production of US vRNA after 24-48h, followed by detection of the structural viral protein p24 (Pomerantz et al., 1990; Seshamma et al., 1992). These cell lines thus represent a model of latency that resembled the early stages of a productive infection, with a block to progression to viral production (Seshamma et al., 1992; Li et al., 1996). Later reports showed that, in fact, HIV-1 persistance in other cell line models of latency, such as J1.1, is characterized by a disproportionate higher US vRNA abundance compared to MS vRNA (Butera et al., 1994). Indeed, analysis of vRNA dynamics in PLWH showed that while US vRNA is detected at low levels in CD4+ T cells, MS vRNA is barely detectable, resulting in the presence of a very high US/MS vRNA ratio, that is maintained even after ex vivo stimulation (Fischer et al., 2002; Hermankova et al., 2003; Yukl et al., 2018). The picture therefore emerges of the presence of blocks at multiple steps in gene expression that include transcriptional initiation and elongation, but also post-transcriptional steps, which result in latency (Lin et al., 2003; Lassen et al., 2004; Yukl et al., 2018).

**Figure 2:**
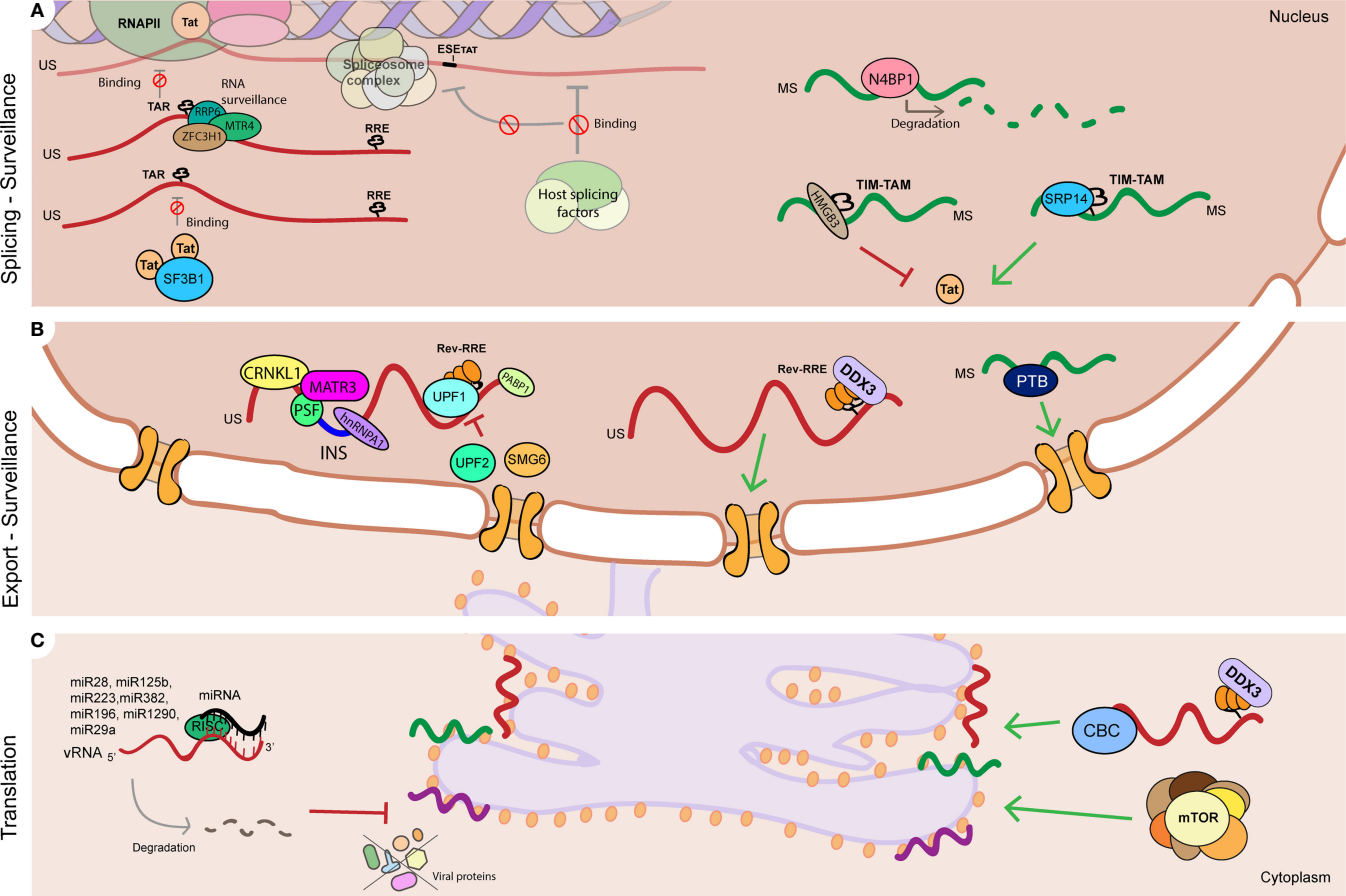
RNA processing mechanisms linked to HIV-1 latency. **(A)** Regulation of HIV-1 vRNA splicing is involved in HIV-1 latency by multiple mechanisms such as: Tat protein levels and activity at the HIV-1 LTR TAR can be controlled by regulating Tat-containing mRNA splicing (ESE-Tat), and action of host factors that prevent Tat-TAR interaction by binding to TAR (SF3B1), and positively (SRP14) or negatively (HMGB3) regulate Tat mRNA processing and translation by binding to TIM-TAM RNA element. Viral RNA processing is also controlled by surveillance mechanisms that prevent Tat-TAR binding in the US vRNA (RRP6, MTR4, ZFC3H1), promote degradation of MS vRNA (N4BP1), and **(B)** regulate US vRNA stability (UPF1, UPF2 and SMG6). **(B)** Nucleocytoplasmic export of HIV-1 vRNA species is tightly regulated during latency: nuclear retention of US vRNA species is promoted by host proteins CRNKL1, MATR3 and PSF, and by binding of host proteins to instability sequences (INS) such as PSF, hnRNPA1 and PABP1. On the contrary, protein DDX3 and PTB positively regulate US vRNA and MS vRNA export respectively, and their absence leads to vRNA nuclear retention. **(C)** Modulation of viral protein synthesis can contribute to HIV-1 latency via: regulation of mTOR complex, DDX3 and CBC complex, and by microRNAs that directly target HIV-1 vRNA and lead to its degradation (miR28, miR125b, miR223, miR383, mimR196, miR1290, miR29a). RNA (vRNA) is represented as a red curved line. Single spliced (SS) 4kb viral RNA is represented as a purple curved line. Multiple spliced (MS) 2kb viral RNA is represented as a green curved line. Red flat-head arrows represent mechanisms reported in literature to promote HIV-1 latency. Green triangle-head arrows represent mechanisms reported in literature to prevent HIV-1 latency. TAR stands for trans-activating response element. RRE stands for Rev response element. INS stands for instability sequence. Red sign ∅ represents inhibition.

Regulation of vRNA splicing impacts the ratio of splicing variants, influencing viral gene expression and hence establishment and reactivation from latency (**Figure 2A**). Splicing of Tat and Rev-containing RNAs is a highly controlled process as their availability is necessary for efficient gene expression. Splicing of Tat-containing RNAs is positively regulated by a specific exonic splicing enhancer (ESE-tat), that is necessary to produce sufficient levels of Tat protein to achieve productive viral replication (**Figure 2A**) (Erkelenz et al., 2015). Polymorphisms in the ESE-tat region that disrupt binding of host splicing factors have a dramatically negative effect on HIV-1 reactivation from latency, confirming that regulation of Tat mRNA splicing is a crucial step in maintenance of latency (Norton et al., 2019). Other post-transcriptional mechanisms have been shown to influence Tat activity and impact HIV-1 reactivation from latency. Kyei et al. showed that splicing factor 3B subunit 1 (SF3B1) interacts with Tat and its presence prevents reactivation of HIV-1 with multiple latency reversing agents (**Figure 2A**) (Kyei et al., 2018). LRAs from the class of histone deacetylase inhibitors, such as Vorinostat, have been described to alter the pattern of splicing variants in cells from PLWH leading to accumulation of MS vRNA in a Tat-dependent manner (Khoury et al., 2018). More recently, it was shown that several RNA binding proteins can regulate Tat RNA processing and translation by binding to an RNA regulatory element (referred to as Tat IRES modulator of tat mRNA (TIM TAM)) present in Tat-containing transcripts. This study showed that host RNA binding proteins SRP14 (splicing regulator) and HMGB3 bind TIM TAM and modulate Tat gene expression and function and can act as positive (SRP14) or negative (HMGB3) regulators of HIV-1 viral production (**Figure 2A**) (Khoury et al., 2021). Recent transcriptomics and proteomics studies have also identified several putative splicing regulators in reactivation of HIV-1 latency (see section 2).

In summary, mechanisms that control HIV-1 latency and latency reactivation include regulation of HIV-1 vRNA splicing by modulating the binding of host spliceosome complex factors to nascent HIV-1 vRNA or by acting on HIV-1 vRNA regulatory elements (Erkelenz et al., 2015; Khoury et al., 2018; Kyei et al., 2018; Yukl et al., 2018; Norton et al., 2019; Telwatte et al., 2019; Khoury et al., 2021).

### 3.2 Nucleocytoplasmic Transport

The 3 classes of HIV-1 vRNA splicing variants, US 9kb vRNA, SS 4kb vRNA and MS 2kb vRNA transcripts, are exported and processed by different mechanisms (**Figure 1**) (Caputi, 2011; Karn and Stoltzfus, 2012; Stoltzfus, 2009). In the early phases of the infection, MS vRNA transcripts are exported *via* canonical NXF1/NXT1-mediated export pathways (Cullen, 2003; Kohler and Hurt, 2007). Rev, encoded by the exported MS vRNA, plays a central role in the nucleocytoplasmic export of US and SS vRNA *via* a CRM1-dependent mechanism (Schwartz et al., 1992; Schwartz et al., 1992). Rev contains a nuclear localization signal (NLS) that facilitates its entry into the nucleus *via* an interaction with Importin B (Henderson and Percipalle, 1997) where it directly targets HIV-1 US and SS vRNAs for export *via* interaction with the trans-acting Rev responsive element (RRE), present within the *env* intron (Malim et al., 1989; Rausch and Le Grice, 2015). Once a sufficient threshold of Rev is achieved in the nucleus, multiple Rev molecules multimerize and bind the RRE (Malim and Cullen, 1991; Rausch and Le Grice, 2015). The resulting Rev-RRE RNP complex recruits host cell protein CRM1 to the nuclear export signal (NES) of Rev, that allows for its export to the cytoplasm (Neville et al., 1997; Askjaer et al., 1998). CRM1 and Rev/RRE form an RNP containing host proteins such as Ran-GTP, components of the nuclear pore complex and other RBPs such as eIF5a (an adaptor for Rev-RRE and CRM1), RNA helicases DDX1 and DDX3 (that facilitate Rev-RRE-Crm1 shuttling) and Sam68 that binds the Rev-RRE-CRM1 RNP promoting its export into the cytoplasm and counteracting inhibiting signals (Ruhl et al., 1993; Yedavalli et al., 2004; Fang et al., 2005; Modem et al., 2005; Modem and Reddy, 2008; Zhou et al., 2009; Monette et al., 2011). Other host proteins [Reviewed in (Luo et al., 2016; Truman et al., 2020)] have also been reported to be required for the regulation and efficient export of Rev-RRE complexes. The HIV-1 US vRNA contains cis-acting AU-rich instability sequences (INS) that regulate mRNA stability, export and translation and are counteracted by Rev (Cochrane et al., 1991; Maldarelli et al., 1991; Schwartz et al., 1992; Olsen et al., 1992; Mikaelian et al., 1996). Although the exact function of INS is yet to be fully understood, they contain AU-rich motifs, a hallmark of unstable RNA elements (Hentze, 1991). Several host proteins such as PABP1, hnRNP A1, PSF and p54 have been found to bind INS and possibly prevent access of the splicing machinery, supporting nuclear retention of INS-RRE containing HIV-1 vRNAs (Black et al., 1996; Afonina et al., 1997; Zolotukhin et al., 2003). Interestingly, recent reports suggest that both US and SS vRNAs can also be exported into the cytoplasm by facilitated diffusion mechanisms irrespective of the transport pathway and that the lack of Rev can be compensated by the overexpression of host proteins such as UPF1 (Ajamian et al., 2015; Chen et al., 2020).

#### 3.2.1 Link to Viral Latency

Viral production is auto-regulated by levels of viral protein Rev. Accumulation of sufficient Rev protein above a certain threshold is strictly necessary for the binding to and export of US and SS vRNA species to the cytoplasm, that encode for the viral genome and structural proteins and other accessory proteins (Pomerantz et al., 1992; Yedavalli and Jeang, 2011a; Truman et al., 2020). In ART-suppressed PLWH, even during clinical latency, a residual level of transcription exists (Lewin et al., 1999; Wiegand et al., 2017). The nuclear retention of vRNA transcripts therefore may contribute to viral latency and it is, hence, heavily enforced by the absence of Rev and Tat, and regulation of MS vRNA species that encode for these proteins is a crucial mechanism for the establishment and maintenance of viral latency. Lassen et al. first showed that HIV-1 MS vRNA transcripts are sequestered in the nucleus during latency, hence resulting in termination of the Tat/Rev positive feedback loop. This proved a defect in export of MS vRNA, preventing translation of Tat and Rev and hence contributing to the maintenance of latency (Lassen et al., 2006). The termination of the Tat-Rev positive feedback loop seemed to be driven by RNA binding proteins that either negatively regulate the export of MS vRNA species or proteins whose absence leads to a block in export. This block in export of MS HIV-1 vRNA species can be reversed by modulating the presence or absence of nucleocytoplasmic export regulators. For instance, overexpression of protein Polypyrimidine tract-binding protein (PTB)1 (**Figure 2B**) leads to increased export of tat-rev containing mRNAs (Lassen et al., 2006), although its exact mechanism of action and contribution to HIV-1 latency remains unclear (Khoury et al., 2021). Still, once the total amount of HIV-1 Tat and Rev increases over a certain threshold, the restored Tat-rev positive feedback loop facilitates transcription, export, and production of viral particles. Thus, viral production in latent cells is negatively regulated by a limited nuclear transport of MS HIV-1 vRNA species and therefore latency can be at least partially maintained by absence of Tat and Rev (Lassen et al., 2004; Lassen et al., 2006).

Similarly, negative regulation of nucleocytoplasmic export of Rev-dependent RNA species prevents efficient gene expression and enforces viral latency. Host proteins PSF, PABP1 and hnRNP A1 act as negative regulators of US vRNA export as they facilitate retention of US vRNA species in the nucleus by binding to instability sequences at introns present in the HIV-1 US vRNA, preventing spliceosome assembly, and prohibiting splicing and export of intron-containing viral mRNA species (**Figure 2B**) (Black et al., 1996; Afonina et al., 1997; Zolotukhin et al., 2003). In particular, Protein PSF has been shown to interact with protein MATR3 and promote nuclear retention of intron-containing viral RNAs, that is reversed upon Rev interaction (Kula et al., 2011; Yedavalli and Jeang, 2011b; Kula et al., 2013). Sarracino et al. showed that PSF/MATR3-mediated retention of US and SS vRNA in the nucleus enforces viral latency as low MATR3/PSF protein levels prevent HIV-1 latency reactivation upon treatment with latency reversing agents (Sarracino et al., 2018). (**Figure 2B**). Spliceosome protein CRNKL1 has been recently implicated in promoting nuclear retention of intron-containing US vRNA species and its depletion leads to improved export of US vRNA (**Figure 2B**) (Xiao et al., 2021; Ne et al., 2022). On the contrary, other proteins can act as positive regulators of US vRNA export and their absence leads to nuclear retention of such species. We have recently shown that pharmacological inhibition of Rev-binding helicase DDX3 leads to nuclear retention of HIV-1 US vRNA and has a role in HIV-1 latency reactivation (Rao et al., 2021).

The need for a threshold of Rev proteins to facilitate export of Rev-containing transcripts that lead to viral production is accentuated by the need for multiple Rev molecules to bind RRE as it has been shown that interaction of a single molecule of Rev with RRE is insufficient for efficient Rev-dependent export (Malim and Cullen, 1991; Pomerantz et al., 1992; Jackson et al., 2016). In addition to its critical role in export of Rev-dependent US and SS vRNA species, Rev has also been implicated in regulating stability of US vRNA and translation of viral proteins, and its role in splicing and spliceosome assembly is speculative (extensively reviewed in (Truman et al., 2020)).

Hence, regulation of nucleocytoplasmic export of HIV-1 vRNA, the ratios of Rev vRNA and viral protein, and nuclear retention of US and MS vRNA species has a direct effect on viral production and promotion and/or maintenance of viral latency (Black et al., 1996; Afonina et al., 1997; Lassen et al., 2006; Sarracino et al., 2018; Xiao et al., 2021; Rao et al., 2021).

### 3.3 RNA Surveillance and Degradation

In eukaryotic cells, RNA surveillance mechanisms in the nucleus and cytoplasm identify and degrade aberrant mRNAs and function as a quality control mechanisms that prevent the accumulation of potentially toxic truncated proteins. In the nucleus, the RNA exosome complex regulates mRNA quality control (reviewed in (Kilchert et al., 2016)). Following export to the cytoplasm, aberrant RNAs are degraded *via* the nonsense-mediated decay (NMD) pathway. Other pathways such as the no-go decay (NGD) pathway, non-stop decay (NSD) and Staufen-Mediated mRNA Decay (SMD) also regulate cytoplasmic RNA quality control. The HIV-1 US vRNA contains large introns, a long UTR and multiple AU-rich instability regions in the 3’UTR (Toro-Ascuy et al., 2016). However, not only does HIV-1 evade RNA quality control, the virus hijacks and recruits host mRNA decay proteins to regulate multiple phases of its own HIV-1 mRNA processing (reviewed in (Toro-Ascuy et al., 2016)). UPF1, the central protein involved in NMD, has been shown to interact with US HIV-1 vRNA, promote in nucleocytoplasmatic transport and translation and is incorporated in virus particles (Ajamian et al., 2008; Ajamian et al., 2015). Knockdown of UPF1 resulted in decreased viral production and a reduction in the infectivity of released particles and overexpression of UPF1 resulted in enhanced viral production (Ajamian et al., 2008). These roles of UPF1 in vRNA metabolism were independent of its roles in NMD and were mediated by its ATP-ase domain. UPF2, SMG6 and UPF3A, other proteins involved in NMD, were found to be negative regulators of HIV-1 gene expression and are excluded from HIV-1 US vRNA RNPs (Ajamian et al., 2015; Rao et al., 2019). Small interference RNA-mediated depletion of UPF2 and SMG6 resulted in enhanced viral replication in primary monocyte-derived macrophages (Rao et al., 2019). Staufen1, involved in SMD, also mediates multiple steps of vRNA processing including vRNA translation, trafficking and assembly (Chatel-Chaix et al., 2004; Dugré-Brisson et al., 2005; Rao et al., 2019).

#### 3.3.1 Link to Viral Latency

Given the distinct roles of NMD proteins in modulating HIV-1 gene expression, follow up work demonstrated that the NMD proteins UPF1, UPF2 and SMG6 also positively (UPF1) or negatively (UPF2, SMG6) influence HIV-1 reactivation from latency by regulating HIV-1 US vRNA stability in latently-infected T cell lines, thereby highlighting a role for the post-transcriptional control of reactivation from latency (**Figure 2B**) (Rao et al., 2018). UPF2 was also found to be enriched in HIV-1 vRNA-expressing cells upon latency reversal (Liu et al., 2020). Proteins involved in nuclear RNA surveillance pathways such as RRP6, MTR4, ZCCHC8 and ZFC3H1 were also found to regulate HIV-1 latency by directly binding to the HIV-1 TAR region thereby occluding the binding of RNAPII to the LTR (**Figure 2A**) (Contreras et al., 2018). A knockdown of these factors resulted in a reactivation of HIV-1 latency in T-cell derived cell lines and infected PBMCs (Contreras et al., 2018). Other cellular proteins have been shown to contribute to HIV-1 latency by direct binding to vRNA and its degradation, such as the RNAse proteins N4BP1 and MALT1 (**Figure 2A**). N4BP1 binds to and degrades HIV-1 US vRNA, repressing HIV-1 gene expression. Inactivation of N4BP1 by induction of MALT1 protein expression leads to HIV-1 reactivation from latency, indicating a role for the N4BP-MALT1 interplay in HIV-1 latency (Yamasoba et al., 2019).

Thus, cellular factors and pathways that control RNA surveillance, stability and degradation of HIV-1 vRNA species are prominently involved in inhibiting viral production and enforcing latency (Ajamian et al., 2015; Contreras et al., 2018; Yamasoba et al., 2019; Liu et al., 2020).

### 3.4 Epitranscriptomic Modifications and RNA Methylation

Viral RNAs, like host cell RNAs, are subjected to epitranscriptomic modifications that regulate their processing, export, and further translation. The most abundant mRNA modification is N6-methyladenosine (m6A), catalyzed by methyltransferase complexes, that occurs mainly in the consensus motif RRACH in the 5 or 3’UTR of the mRNA (Riquelme-Barrios et al., 2018; Jiang et al., 2021). Addition of an N6-methyladenosine is catalyzed by writers METTL3, METTL14 and WTAP and the methyl group can be also removed by erasers such as ALKBH5 and FTO (Riquelme-Barrios et al., 2018; Jiang et al., 2021). The m6A addition to the mRNA are recognized by the reader proteins including the predominantly nuclear YTHDC1 and the cytoplasmic YTHDC2, YTHDF1, YTHDF2 and YTHDF3 proteins that regulate mRNA processing, export, stability and translation of m6a-containing mRNAs (Meyer and Jaffrey, 2014; Jiang et al., 2021). In context of the HIV-1 US vRNA, methylation of nascent vRNA transcripts is a critical step in HIV-1 vRNA metabolism (reviewed in (Riquelme-Barrios et al., 2018)). Lichinchi et al. reported that the viral RNA contains multiple m6a editing sites, including at the RRE, and knockdown of methyl eraser ALKBH5 resulted in increased abundance of methylated HIV-1 US vRNA, enhanced Rev-RRE interaction and vRNA export, and increased viral gene expression (Lichinchi et al., 2016) The role of m6A methylation in Rev-RRE mediated vRNA export is, however, controversial, as other studies failed to detect presence of m6A editing sites at the RRE (Tirumuru et al., 2016; Kennedy et al., 2017; Lu et al., 2018). Besides, N6-adenosine writer (METTL3, METTL14) and reader proteins (YTHDF1-3) have been shown to modulate HIV-1 viral replication by affecting Gag expression (METTL3, METTL14) and decreasing viral genomic RNA and inhibiting reverse transcription (Tirumuru et al., 2016; Lu et al., 2018). The m6A reader YTHDF proteins also influence HIV-1 US vRNA expression, although they are reported to have conflicting effects on HIV-1 viral gene expression that remain to be characterized (Kennedy et al., 2017; Lu et al., 2018). These proteins are also implicated in the degradation of incoming genomic vRNA (Tirumuru et al., 2016). More recently it has been shown that m6A addition to HIV-1 vRNA also influences its splicing and stability and affects gene expression mediated by YTH family proteins YTHDF1 and YTHDF2, which bind and stabilize vRNAs, regulate alternative splicing and overall increase HIV-1 gene expression (Tsai et al., 2021). HIV-1 vRNAs can also be regulated by addition of other modifications such as 5-methylcytosine (m5C). Courtney et al. showed in their recent study that addition of m5C to the HIV-1 US vRNA is mediated by writer NSUN2 and its absence influences alternative splicing, export and translation of Gag-containing transcripts, and leads to decreased gene expression (Courtney et al., 2019). Interestingly, HIV-1 infection also causes changes in the epitranscriptomic profile of host cell RNAs and hence influences their overall downstream processing (Cristinelli et al., 2021; Zhang et al., 2021).

#### 3.4.1 Link to Viral Latency

Although no direct link of epitranscriptomic modifications of the HIV-1 vRNA to the maintenance of latency has been reported so far, the multiple roles on RNA metabolism mediated by epitranscriptomic modifications would warrant further investigation of the roles of the methyl writers, erasers and readers and their contribution to viral latency.

### 3.5 Translation

The translation of viral proteins and viral assembly, followed by budding and virion maturation, are the last steps in the HIV-1 life cycle in order to produce infectious viral particles (**Figure 1**). Similar to host mRNAs, HIV-1 vRNAs contain a 5’ cap structure necessary for translation initiation (Ohlmann et al., 2014; Guerrero et al., 2015). In the case of US vRNA, its fate, whether it serves as genomic vRNA or template for translation initiation, is hypothesized to depend on the presence of a secondary structure in the 5’ UTR that can present two conformations: branched multiple hairpins (BMH) that favors viral RNA encapsidation, or long distance interaction (LDI) that favors translation initiation (Huthoff and Berkhout, 2001; Berkhout et al., 2002; Ooms et al., 2004). The TAR structure in the 5’UTR has been shown to pose a barrier to cap-dependent translation initiation due to the presence of secondary RNA structures. This is why RNA helicases and other host proteins such as such as TRBP, Staufen1 or helicase DDX3 play an important role in preserving an efficient translation of the HIV-1 vRNA. Translation of HIV-1 US vRNA can also be initiated *via* a cap-independent mechanism that involves an internal ribosome entry site (Monette et al., 2013). The HIV-1 US vRNA contains two IRES sequences, one is present in the 5’UTR, and the second one is present within the Gag region. Translation initiation from IRES sequences is crucial in later stages of the viral cycle, where cap-dependent translation initiation is impaired (Amorim et al., 2014). In addition, HIV-1 restriction factors can act by modulating the synthesis of viral proteins *via* distinct mechanisms, such as Schalfen protein SLFN11, that prevents vRNA translation by codon usage-based inhibition in response to interferon induction (Li et al., 2012).

Translation of viral proteins is also highly regulated by small non-coding RNAs, such as microRNAs. For example, miR29a targets HIV-1 US vRNA leading to translational repression, miR133b, miR138-5, miR326, miR149-5p and miR92a-3p have been shown to reduce HIV-1 replication and miR125 expression negatively correlates with HIV-1 infection (Houzet et al., 2012; Mantri et al., 2012). MicroRNAs can also negatively control HIV-1 gene expression and indirectly targeting essential co-factors for HIV-1 replication (reviewed in (Balasubramaniam et al., 2018; Sadri Nahand et al., 2020)) As so, there is ongoing discussion whether the HIV-1 genome encodes functional microRNAs (Ouellet et al., 2013; Balasubramaniam et al., 2018; Sadri Nahand et al., 2020). A recent report described the presence of microRNA miR-n367, found in the *nef* region of the HIV-1 genome, that efficiently targets and inhibit Nef protein synthesis, affecting overall HIV-1 gene expression (Omoto et al., 2004).

#### 3.5.1 Link to Viral Latency

Although the link of HIV-1 vRNA translation and latency has remained under-investigated, several reports implicate translational inhibition in contributing to HIV-1 latency. Only up to 10% of the vRNA+ cells are able to produce viral protein even after maximum reactivation of latently-infected cells, indicating that viral protein synthesis is strongly inhibited during latency (Grau-Exposito et al., 2019; Sannier et al., 2021). The Verdin group first reported the role of mTOR, the master regulator of host cell translation (Nandagopal and Roux, 2015), in HIV-1 gene expression and latency and showed that mTOR1 and mTORC2 complex inhibition leads to suppression of HIV-1 transcription and reactivation from latency in a Tat-independent manner (**Figure 2C**) (Besnard et al., 2016). This effect seems to be mediated by mTOR pathway inhibitor proteins, such as TSC1 and DEPDC5 (Jin et al., 2018). One other protein potentially involved in HIV-1 latency is host restriction factor SLFN11, that, as mentioned before, prevents the synthesis of viral proteins and has been found to correlate with HIV-1 persistance in ART-suppressed PLWH (Abdel-Mohsen et al., 2015). Besides, as discussed above, viral protein Rev and its co-factors, for example DDX3 or CBP80/20, have a very prominent role in HIV RNA translation (**Figure 2C**) (Arrigo and Chen, 1991; Perales et al., 2005; Soto-Rifo et al., 2012; Frohlich et al., 2016; Garcia-de-Gracia et al., 2021). Thus, regulation of Rev production and DDX3-mediated translation of viral RNAs are important mechanisms involved in latency at this level.

Cellular microRNAs have also been described to have a role in HIV-1 latency (recently reviewed in (Heinson et al., 2021)). Several micro RNAs have been described to directly target HIV-1 vRNA and modulate its regulation (**Figure 2C**). MicroRNAs miR28, miR125b, miR223 and miR382 target motifs in the 3’ of HIV-1 vRNA and have a role in the maintenance of latency as their inhibition by antisense nucleotides resulted in reactivation of HIV-1 from latency (Huang et al., 2007). Also, inhibition of miR196 and miR1290 that target the 3’ untranslated region of the HIV-1 transcript, led to restored HIV-1 replication, suggesting a role in promoting latency (Wang et al., 2015). MicroRNA miR29a interacts with the 3´UTR of HIV-1 vRNA and correlates inversely with HIV-1 replication in HIV-1 infected latent cell line models and is thus linked to maintenance of HIV-1 latency (Patel et al., 2014). MicroRNAs are also thought to be associated with HIV-1 latency in an indirect manner by targeting and preventing translation of host factor proteins involved in transcription silencing, survival and by controlling the availability of co-factors such as cyclin T1, PCAF or TRIM32 (Triboulet et al., 2007; Chiang et al., 2012; Ruelas et al., 2015; Yang et al., 2015; Yin et al., 2015). Given their role in the establishment and maintenance of latency, microRNAs could potentially be used as a therapeutic option to reverse or reinforce HIV-1 latency (Section 5.3).

To conclude, present literature illustrates that inhibition of viral protein synthesis contributes to HIV-1 latency (Grau-Exposito et al., 2019; Sannier et al., 2021). While the mechanisms behind this process require further investigation, several reports highlight the role of factors involved in host mRNA translation and microRNA-mediated vRNA degradation (Abdel-Mohsen et al., 2015; Besnard et al., 2016; Heinson et al., 2021).

## 4 Implications for Cure Strategies

Currently, two HIV-1 curative approaches have been explored to target the latent virus: “shock-and-kill” or “block-and-lock” (Deeks et al., 2021). The first aims to reverse HIV-1 latency (shock) followed by promoting viral clearance of reactivated cells (kill) (Deeks, 2012; Deeks et al., 2021). The second lesser investigated approach pursues permanent silencing of HIV-1 viral production (block-and-lock) (Ahlenstiel et al., 2020; Deeks et al., 2021). How the post-transcriptional regulation of HIV-1 could affect these strategies remains largely underexplored. The current spectrum of latency reversing agents under investigation, either reactivate or permanently enforce HIV-1 latency by targeting pathways involved in HIV-1 transcription initiation or elongation (Stoszko et al., 2019). The development of therapeutic interventions that interfere with HIV-1 RNA processing is an emerging field, also in context of HIV-1 cure. In fact, two recent studies have reported that the pharmacological modulation of HIV-1 gene expression by targeting HIV-1 vRNA splicing silences HIV-1 gene expression (Yeh et al., 2020), and can lead to a temporal reduction in total HIV-1 DNA (Moron-Lopez et al., 2021). Pharmacological targeting of the mTOR pathway using small molecule inhibitors, is also being explored for their potential in targeting and decreasing HIV-1 DNA+ in CD4+ T cells from PLWH *in vivo* (Stock et al., 2014; Henrich et al., 2021). These studies are proof-of-concept that targeting RNA processing pathways is a promising approach to be considered for inclusion in future cure strategies.

With regards to the “shock” of the shock-and-kill strategy, as mentioned before, almost all known LRAs target LTR-driven transcription by antagonizing chromatin-mediated repression, or inducing transcription initiation or elongation, for example by triggering NFκB-mediated transcription activation (Stoszko et al., 2019). LRAs tested in clinical studies have shown negligible effect in depleting the viral reservoir (Routy et al., 2012; Elliott et al., 2014; Sogaard et al., 2015; Tsai et al., 2016), likely due to poor production of viral antigens that can be recognized by the immune system. This demonstrates that the “kill” aspect of this strategy requires more attention and could be benefited from more efficient antigen presentation. Numerous studies have shown that, while current LRAs are able to efficiently promote transcription of HIV-1 and increase cell-associated US vRNA, they fail to increase production of spliced vRNA transcripts (Telwatte et al., 2019; Moron-Lopez et al., 2019; Sannier et al., 2021; Zerbato et al., 2021). However, it is the intracellular induction of MS vRNA species, but not US vRNA, that has been shown to correlate with increase in viral protein production and plasma HIV-1 RNA (Sannier et al., 2021; Zerbato et al., 2021), thereby highlighting the importance of effective vRNA splicing for viral production and antigen presentation. Therefore, transcriptional activation alone is not sufficient to induce spliced vRNA species and viral protein production since co and post-transcriptional blocks are not targeted by current LRAs. Robust viral protein production is, however, necessary for antigen presentation and further recognition by cytotoxic immune cells that promote immune cell killing (ICK). Hence, novel therapeutic combinations that lead to release of post-transcriptional blocks and enhance production of MS vRNA species and viral protein production is likely crucial in order to achieve killing of reactivated infected cells by extrinsic cell mechanisms.

For the “kill” of the shock-and-kill strategy, the elimination of HIV-1 reservoirs could also be accomplished by triggering intrinsic cell death (ICD) mechanisms that promote selective apoptosis of HIV-1 infected cells by the pharmacological targeting of apoptosis pathways (Chen et al., 2022). Recent reports have highlighted the role of intron-containing vRNA accumulation in stimulating the antiviral host cell response, which can potentially trigger apoptosis pathways in HIV-1 infected cells selectively (Li et al., 2016; Garcia-Vidal et al., 2017; Fong et al., 2017; Rao et al., 2021). The presence of intron-containing HIV-1 vRNA is reported to trigger innate immune signaling responses *via* the RIG-I and MAVS pathways in dendritic cells, macrophages and CD4+ T cells (Berg et al., 2012; Akiyama et al., 2018; McCauley et al., 2018). These responses are designed to sense viral RNA, stimulate IFN signaling and lead the cell to apoptosis (Balachandran et al., 2000; Chawla-Sarkar et al., 2003). Recent studies have also highlighted the role of nuclear viral RNA sensors, including innate immune sensor RIG-1, previously thought to be only present in the cytoplasm (Liu et al., 2018). However, during HIV-1 infection antiviral responses are dampened by viral proteins that counteract innate immune responses (Chintala et al., 2021). Manipulation of the pathways that regulate host-virus pathogenicity can shift the balance towards immune signaling and cell death (Fong et al., 2017). Following such approach, modulating RNA metabolism pathways so as to promote post-transcriptional blocks and regulate mistrafficking of vRNA is desired in order to accumulate sufficient vRNA levels that can activate innate immune signaling pathways. For example, our group has recently reported that pharmacological inhibition of DDX3 leads to latency reversal and prevents export and translation of HIV-1 US vRNA. Consequently, DDX3 inhibition causes accumulation of HIV-1 US vRNA species that can trigger the innate antiviral signaling pathway and leads to selective apoptosis of infected cells (Rao et al., 2021).

What does this all mean for a shock-and-kill cure approach? In short, what we consider a surrogate for viral reactivation and how RNA metabolism pathways can be manipulated to achieve viral clearance ultimately depends on the chosen approach and desired outcome. What biomarkers we use when designing and analyzing HIV-1 cure interventions, whether that is US vRNA, MS vRNA, or viral protein, is a subject of continuous debate and has been thoroughly discussed by others (Pasternak and Berkhout, 2018; Pasternak and Berkhout, 2021). When evaluating an effective “shock” and latency reversal, both US and MS vRNA readouts should be considered as markers for latency reversal and viral production. While MS vRNA is a better surrogate for viral reactivation of the replication-competent reservoir and viral protein production (Zerbato et al., 2021), the US vRNA is important for triggering the innate immune signalling and apoptosis pathways (Rao et al., 2021). Therefore when pursuing an ICK approach for the “kill” in shock-and-kill, MS vRNA is a relevant readout. Whereas in an ICD approach, US vRNA needs to be quantified.

In an ICK approach, pharmacological activation of HIV-1 transcription by LRAs can be accompanied by approaches that target multiple levels of HIV-1 post-transcriptional regulation to achieve MS vRNA induction and viral protein expression for efficient antigen presentation. For example, by targeting mechanisms that promote splicing, export and translation of vRNA, reverting nuclear retention of MS vRNAs or preventing degradation of MS vRNA species. Induction, export and translation of MS vRNAs will result in a restored Tat/Rev feedback loop that aids in LTR-driven transcription (Tat) and increased levels of Rev can promote processing and translation of Rev-containing transcripts, including Env-coding RNAs, that encode for structural proteins, and Gag protein, necessary for viral particle assembly.

In an ICD approach, manipulating RNA metabolism pathways that can inhibit splicing or translation, or promote export of intron-containing HIV-1 vRNA is desired in order to accumulate sufficient levels that can activate innate immune signaling pathways and render the cell sensitive to apoptosis. Promoting nuclear accumulation of intron-containing vRNA by modulating Rev-dependent export can also lead to activation of nuclear innate immune sensors. Other studies have also shown that epigenetic modulation of the HIV-1 US vRNA can trigger innate immune responses, which can be exploited in order to promote selective cell death (Chen et al., 2021). This is also crucial in order to confer selectivity, as uninfected bystander cells will not be pushed towards apoptosis as absence of HIV-1 vRNA prevents induction of antiviral pathways.

Taken together, the multiple layers of HIV-1 RNA regulation and intricate post-transcriptional pathways that enforce latency may complicate the picture of cure approaches that rely on modulation of vRNA processing. Still, we are optimistic that modulation of post-transcriptional mechanisms that influence the ratio, accumulation or downstream processing of HIV-1 vRNAs is a promising approach to be considered in future fundamental and translational cure-based studies and further research into HIV-1 RNA biology is needed in order to tackle the obstacles currently faced in eliminating HIV-1 reservoirs.

## 5 Therapeutic Targeting of HIV-1 RNA Metabolism Pathways

Throughout this review we have extensively discussed HIV-1 vRNA metabolism and the role of RNA processing pathways in regulating HIV-1 gene expression and latency. Therapeutic modulation of RNA metabolism offers an interesting new angle in context of strategies aimed at reducing or eliminating the viral reservoir. Targeting RNA processing pathways has so far been explored in order to restrict HIV-1 replication, but these strategies have been mainly discussed for their role as antiviral therapeutics (Wong R et al., 2013; Dlamini and Hull, 2017). Repurposing these therapeutics for their use in cure strategies is an interesting approach that we believe merits further investigation.

**Figure 3:**
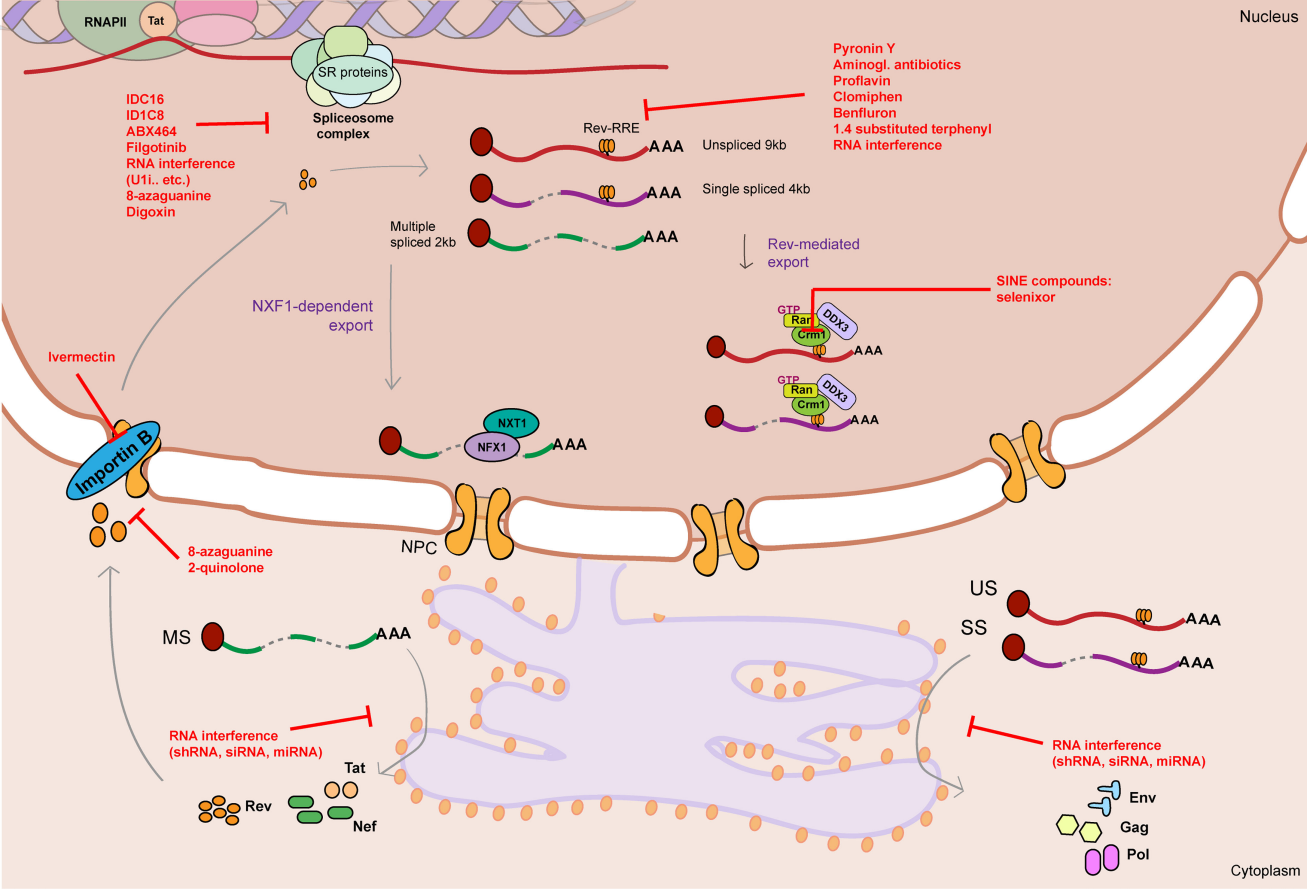
Graphical summary of therapeutics that target HIV-1 RNA metabolism. HIV-1 RNA metabolism can be therapeutically targeted by drugs/therapeutics that interfere with splicing (IDC16, ID1C8, ABX464, Filgotinib, RNA interference therapeutics, 8-azaguanine, Digoxin), Rev-RRE binding (Pyronin Y, Aminoglycosid antibiotics, proflavine, Clomiphen, Benfluron, 1.4 substituted terphenyl, RNA interference therapeutics), Rev-mediated export (CRM1 inhibitors: SINE compounds), Rev nuclear import (Ivermectin, 8-azaguanine, 2-quinolone) and translation of viral RNA (RNA interference therapeutics). Unspliced 9kb viral RNA is represented as a red curved line. Single spliced 4kb viral RNA is represented as a partially cut purple curved line. Multiple spliced 2kb viral RNA is represented as a partially cut green curved line. Red flat-head arrows represent therapeutic targets. RRE stands for Rev response element. NPC stands for nuclear pore complex.

The spectrum of therapeutics so far tested that target RNA metabolism mechanisms in regulation of HIV-1 gene expression mainly focus on three processes: HIV-1 alternative splicing, Rev-mediated RNA export and RNA interference (**Table 1** and **Figure 3**).

**Table 1:** Summary of therapeutics that target and modulate HIV-1 RNA processing pathways.

Therapeutic	Target (pathway)	Clinical status	Ref
IDC16	Alternative splicing: Inhibition of ESE-dependent splicing by SR (AF/SF2) binding	Pre-clinical	(Bakkour et al., 2007)
ID1C8	Alternative splicing: Inhibition splicing regulator SRSF10	Pre-clinical	(Cheung et al., 2016; Shkreta et al., 2017)
ABX464	Alternative splicing, Rev-mediated export and CBC interaction	FDA/EMA-approved	(Campos et al., 2015; Vautrin et al., 2019; Moron-Lopez et al., 2021)
GPS491	Alternative splicing: modulation SR proteins	Pre-clinical	(Dahal et al., 2022)
Filgotinib	Alternative splicing and JAK-STAT pathway	FDA/EMA-approved	(Yeh et al., 2020)
Digoxin	Alternative splicing: modulation SR proteins	FDA/EMA-approved	(Wong et al., 2013)
Pyronin Y	Nucleocytoplasmic export: Rev-RRE binding	Pre-clinical	(Schröder et al., 1993)
Aminoglycoside antibiotics	Nucleocytoplasmic export: Rev-RRE binding	FDA/EMA-approved	(Nishizono and Nair, 2000)
Aromatic heterocyclic compounds, Proflavin	Nucleocytoplasmic export: Rev-RRE binding	FDA/EMA-approved	(DeJong et al., 2003)
Clomiphen	Nucleocytoplasmic export: Rev-RRE binding	FDA/EMA-approved	(Prado et al., 2016)
Benzofluorenone (Benfluron)	Nucleocytoplasmic export: Rev-RRE binding	Pre-clinical	(Prado et al., 2018)
1,4-substituted terphenyl compounds	Nucleocytoplasmic export: Rev-RRE binding	Pre-clinical	(Medina-Trillo et al., 2020)
Leptomycin B	Nucleocytoplasmic export: Rev-CRM1	FDA/EMA approved	(Wolff et al., 1997)
SINE: Selenixor	Nucleocytoplasmic export: CRM1 inhibitor	FDA/EMA approved	(Daelemans et al., 2002; Boons et al., 2015; Daelemans et al., 2015)
Ivermectin	Nucleocytoplasmic export: Importing αβ inhibitor	FDA/EMA approved	(Wagstaff et al., 2012)
8-azaguanine	Alternative splicing and nucleocytoplasmic export	Pre-clinical	(Wong RW et al., 2013)
2-quinolone	Nucleocytoplasmic export: Rev translocation	Pre-clinical	(Wong RW et al., 2013)
RNA interference	Alternative splicing (U1i) and regulation of HIV-1 restriction factors (miRNA)	Pre-clinical	(Zhang, 2009; Brosseau et al., 2014; Swaminathan et al., 2014; Del Corpo et al., 2019)

### 5.1 Targeting HIV-1 Alternative Splicing

Regulation of alternative splicing in context of HIV-1 gene expression mainly depends on the action of host factors such as SR and hnRNP proteins that bind, respectively, to exonic splicing enhancer and silencer motifs on the HIV-1 vRNA and modulate splicing efficiency (Caputi et al., 1999; Jablonski and Caputi, 2009; Ohe and Hagiwara, 2015; Dlamini and Hull, 2017). Although SR and hnRNP proteins are part of the host cell splicing machinery, the specificity of HIV-1 towards certain classes and their essential role in efficient viral production make SR proteins an attractive target in the search of novel HIV-1 RNA processing modulating drugs. Therapeutic targeting of HIV-1 alternative splicing can either pursue release or suppression of HIV-1 molecular blocks. Repurposing inhibitors of HIV-1 splicing for cure research becomes an interesting approach to be considered for “block-and-lock” cure approaches aimed at permanently silencing HIV-1 viral production (Yeh et al., 2020; Moron-Lopez et al., 2021), and it might also be relevant in “shock-and-kill” strategies that emphasize accumulation of intron-containing vRNA, which in turn activates innate immune pathways and selectively sensitizes the cell to apoptosis (Berg et al., 2012; Li et al., 2016; Fong et al., 2017; Rao et al., 2021) (see section 4). As so, current cure approaches aimed at inducing HIV-1 transcription *via* latency reversal agents may also be combined with therapeutics that promote HIV-1 splicing and accumulation of MS vRNA, that can in turn restore the Tat/Rev feedback loop and hence fuel transcription, export and translation of vRNA species encoding immunogenic structural proteins.

Multiple small molecules have been described over the last few decades that hinder HIV-1 splicing, mostly by targeting host SR proteins and modulating their phosphorylation status, preventing their role in spliceosome assembly, or inhibiting their binding to the nascent RNA (Bakkour et al., 2007; Ohe and Hagiwara, 2015; Dlamini and Hull, 2017). For example, Bakkour and colleagues performed a large scale screening of putative compounds able to inhibit ESE-dependent splicing by targeting specific SR proteins, preventing spliceosome assembly and leading to HIV-1 splicing inhibition (Bakkour et al., 2007). Tetracyclic indole derivative IDC16 and 1C8 showed significant reduction in HIV-1 replication by directly targeting SR protein ASF/SF2 and SRSF10, respectively, and hence negatively affecting alternative splicing of HIV-1 vRNA (Bakkour et al., 2007; Cheung et al., 2016; Shkreta et al., 2017). Because of the putative *in vivo* adverse effects of these compounds, other derivatives were synthesized that would potentially be more target-specific and have less side effects. The most potent compound was ABX464, that inhibits HIV-1 vRNA splicing but also interferes with cap-binding proteins and Rev-dependent export (Campos et al., 2015; Vautrin et al., 2019), and has recently been shown to temporarily decrease Total HIV-1 DNA in ART-suppressed PLWH (Moron-Lopez et al., 2021). The authors hypothesize that ABX464 could be able to induce aberrant HIV-1 transcripts that can be recognized by the immune system, leading to selective killing of infected cells (Moron-Lopez et al., 2021). More recently, an anti-HIV-1 stilbene quinolone (GPS491) has been shown to modulate the abundance and function of host SR proteins, hence altering the HIV-1 splicing pattern and inhibiting the HIV-1 life cycle (Dahal et al., 2022). Lastly, the design of drug screens coupled with transcriptomic analysis can broaden the range of HIV-1 splicing targeting compounds by multiple other mechanisms. Such was the case of FDA-approved JAK1 inhibitor Filgotinib, that has recently been reported to also suppress HIV-1 splicing and prohibit HIV-1 gene expression in cells obtained from PLWH (Yeh et al., 2020).

Although there has been less research development into the identification of agents able to release HIV-1 splicing blocks, future studies should deepen into discovery and repurposing of agents that act on the host cell alternative splicing mechanisms employed by the virus during vRNA processing. For instance, HIV-1 splicing blocks can potentially be released by altering the function of hnRNP proteins that bind inhibitory exonic splicing silencers (Carabet et al., 2019; Solano-Gonzalez et al., 2021) or inhibit surveillance mechanisms that promote vRNA degradation (Durand et al., 2007; Popp and Maquat, 2015). Another possibility to enhance HIV-1 splicing is by modulating splicing site usage by small molecules or RNA interference therapeutics (see Section 5.3). It is worth noting that changes in the pattern of the tightly-regulated splice site use on the HIV-1 vRNA template can alter the ratios of US/MS vRNAs to suboptimal levels, that will, in fact, result in overall inhibition of HIV-1 gene expression. For example, small molecule Digoxin promotes oversplicing and causes an overall increase in total MS vRNA, but also alters the splicing site usage within the MS vRNA and causes a significant decrease in Rev-containing MS vRNA and Rev protein, that eventually results in inhibition of HIV-1 viral production (Wong et al., 2013). Other compounds, such as 8-azaguanine, are also able to promote oversplicing and cause an increase in MS vRNA, but also target and inhibit Rev nuclear import, which prevents export of Rev-dependent vRNA and production of viral structural proteins (Wong RW et al., 2013).

In conclusion, vRNA alternative splicing is a dynamic process that can be potentially utilized as therapeutic alternative. The design of HIV-1 RNA biology targeting strategies, however, would need to address the the intricate mechanisms that regulate the vRNA/protein equilibrium.

### 5.2 Targeting HIV-1 RNA Nucleocytoplasmic Export

Given its essential role in viral protein translation, vRNA nucleocytoplasmic export also represents an attractive target to modulate HIV-1 vRNA metabolism. Export and translation of HIV-1 US and SS vRNA relies greatly on the interplay between Rev and the RRE motif in the vRNA (Rausch and Le Grice, 2015). Inhibition of Rev by small molecules has been studied for decades as potential therapeutics to inhibit viral production (Wong R et al., 2013). The use of small molecules in cure studies that target viral proteins such as Rev or Rev-RRE interaction is promising because of their potential high specificity and low toxic side effects. In addition, inhibition of Rev-mediated vRNA export has a very strong effect on silencing HIV-1 gene expression and can present a new therapeutic angle in block-and-lock cure approaches (Wong R et al., 2013). Complementary to latency reactivation-based cure interventions, accumulation of viral RNA by inhibiting export and promoting nuclear retention of vRNAs can potentially trigger nuclear innate immune signaling pathways and lead to latency reversal and induced cell death in latently infected cells (see section 4) (Berg et al., 2012; Li et al., 2016; Fong et al., 2017; Rao et al., 2021).

Initially purposed as possible antiretroviral compounds, a variety of drugs have been identified that efficiently alter Rev-RRE binding and lead to inhibition of viral production. Early studies on pharmacological disruption of Rev-RRE interaction by use of small molecules unveiled RNA intercalating drugs such as Pyronin Y, aminoglycoside antibiotics and aromatic heterocyclic compounds that bind to the RRE and prevent Rev-dependent export (Schröder et al., 1993; Nishizono and Nair, 2000; DeJong et al., 2003). In a screen reported by Prado et al., several FDA-approved compounds were discovered to inhibit Rev-RRE binding and alter HIV-1 gene expression, including Clomiphen, an estrogen receptor modulator used for the treatment of infertility in women (Prado et al., 2016). The same group subsequently described another compound, benzofluorenone or Benfluron, that targets Rev-RRE binding by binding to the RRE and inhibits viral gene expression (Prado et al., 2018). Recently, a drug discovery study reported the design of the multi-target small molecule 1,4-substituted terphenyl compounds that inhibit HIV-1 transcription and Rev-dependent export by binding to the RRE element (Medina-Trillo et al., 2020). Nevertheless, while some of these drugs are very effective and sensitive, the relative specificity and possible secondary binding targets (i.e. non-HIV RNAs) of these small compounds is, to our knowledge, poorly described. Ideally, strategies that pursue reservoir elimination and cause limited to no toxicity would benefit from availability of drugs that target vRNA or proteins exclusively.

Complete functionality of Rev depends greatly on its ability to translocate between the cytoplasm and the nucleus, a process mediated by the action of host cell proteins, such as CRM1 and Importin B. The antibiotic leptomycin B was the first compound described to interfere with Rev-CRM1 mediated export of HIV-1 vRNA and inhibit HIV-1 gene expression (Wolff et al., 1997). Later, other compounds called selective inhibitors of exportin-1 (CRM1) nuclear export (SINE) have been synthetized and extensively characterized for their role in suppressing HIV-1 replication, including FDA-approved SINE compound selinexor (Daelemans et al., 2002; Boons et al., 2015; Daelemans et al., 2015). In addition, inhibition of Importin-mediated nuclear import by compound Ivermectin disrupts Rev nuclear import and hence prevents export of Rev-dependent vRNA species, resulting in suppression of HIV-1 replication (Wagstaff et al., 2012). Other compounds, such as 8-azaguanine and 2-quinolone, can also inhibit Rev translocation to the cytoplasm, or lead to decrease in Rev production, resulting in accumulation of intron-containing vRNA in the nucleus (Wong RW et al., 2013). Although some of these drugs, such as Selenixor, are approved by drug administrations, their use in high doses causes reported severe side effects (Grade ≥ 3) in a considerable percentage of participants (Neupane et al., 2021). Their potential use and associated toxicity at lower doses and in combination with other drugs is yet to be studied.

Less is known about therapeutics that enhance Rev-mediated export and promote translation of vRNAs. Promoting vRNA export and viral protein production is of interest in shock-and-kill approaches that aim at reversing post-transcriptional blocks and enhance immune-mediated clearance (see Section 4). Lastly, viral RNA export is influenced by epitranscriptomic modifications of the vRNA. Inhibitors that regulate m6a methylation of viral RNAs would also be interesting to investigate in context of HIV-1 curative approaches since epitranscriptomic modifications influence viral gene expression and modulate antiviral immune responses. For example, activators of the methyl writers could be utilized to promote viral gene expression and be used as latency reversing agents (Selberg et al., 2021).

### 5.3 RNA Interference

RNA interference therapeutics are also a promising approach to target and eliminate HIV-1 infected cells *via* gene therapy strategies (reviewed in (Scarborough and Gatignol, 2017)). Several steps of HIV-1 replication can directly be inhibited by delivery of antiviral RNAs (shRNAs or miRNAs), RNA decoys or aptamers that directly target the LTR, Tat, Rev or Gag proteins, or secondary RNA structures such as TAR or RRE. Alternative splicing of HIV-1 vRNAs can be modulated by blocking splicing sites, causing interference with binding of the spliceosome complex and other co-factors required for efficient splicing. Masking of a specific splicing site can be achieved by complementary antisense RNA sequences (Brosseau et al., 2014). Binding of these sequences to the specific ss-containing motif in the HIV-1 vRNA prevents the access of the splicing machinery leading to splicing inhibition (Brosseau et al., 2014). One example is targeting of the U1 snRNA by U1 interference RNAs. These U1i RNAs are designed to interfere with polyadenylation or enhance splicing of HIV-1 RNA, and they are able to efficiently inhibit HIV-1 replication (Del Corpo et al., 2019). As well, antisense RNA sequences can be found in the form of peptide-nucleotide fused molecules, in which the antisense RNA is fused to a protein involved in splicing regulation or inhibition. A similar approach is the use of bi-functional oligonucleotides that include a target-specific antisense RNA and a second oligonucleotide whose function is to recruit proteins or RNA-protein complexes that interfere with splicing (Brosseau et al., 2014).

Up or downregulation of microRNAs that target specific proteins involved in HIV-1 replication can be used as a potent therapeutic tool for promotion or inhibition of HIV-1 gene expression (Zhang, 2009; Swaminathan et al., 2014). Antisense oligonucleotides that inhibit the effect of miRNA have already been used in context of other diseases, such as Hepatitis C Virus infection in which its replication can be inhibited by targeting mi-R122 (Janssen et al., 2013). However, given their known pleiotropic effects and limitations to the delivery methods available, the use of microRNAs as a therapy is debatable (Swaminathan et al., 2012). Moreover, modulating miRNA as a potential therapy in HIV-1 cure studies has been challenged recently as it has been shown that inhibition of miRNAs previously identified to have a role in latency did not lead to latency reactivation (Lopez-Huertas et al., 2019).

## 6 Conclusion

The role of RNA metabolism in HIV-1 latency has been relatively under-studied, both from a fundamental and a translational perspective. In this review, we extensively discuss how co- and post-transcriptional events are major regulators of HIV-1 gene expression and can also contribute to viral latency. Investigating these pathways is essential to further our current knowledge on viral latency regulation and may be very lucrative in finding novel therapeutic targets that will advance HIV-1 curative strategies.

## Author Contributions

All authors contributed to the writing and revision of the manuscript.

## Funding

TM received funding from the European Research Council (ERC) under the European Union’s Seventh Framework Programme (FP/2007-2013)/ERC STG 337116 Trxn-PURGE, Dutch Aidsfonds grant 2014021, Health Holland grants LSHM19100-SGF and EMCLSH19023, ZonMW grant 40-44600-98-333, and Erasmus MC mRACE research grant. RC received funding from Dutch Aidsfonds Small Grant 2020 P-60603. SR received funding from Dutch Aidsfonds grant P-53302 and Gilead Research Scholars program for HIV.

## Conflict of Interest

The authors declare that the research was conducted in the absence of any commercial or financial relationships that could be construed as a potential conflict of interest.

## Publisher’s Note

All claims expressed in this article are solely those of the authors and do not necessarily represent those of their affiliated organizations, or those of the publisher, the editors and the reviewers. Any product that may be evaluated in this article, or claim that may be made by its manufacturer, is not guaranteed or endorsed by the publisher.

## References

[B1] Abbink T. E. Berkhout B. (2008). RNA Structure Modulates Splicing Efficiency at the Human Immunodeficiency Virus Type 1 Major Splice Donor. J. Virol. 82 (6), 3090–3098. doi: 10.1128/JVI.01479-07 18160437 PMC2258995

[B2] Abdel-Mohsen M. Wang C. Strain M. C. Lada S. M. Deng X. Cockerham L. R.. (2015). Select Host Restriction Factors Are Associated With HIV Persistence During Antiretroviral Therapy. AIDS 29 (4), 411–420. doi: 10.1097/QAD.0000000000000572 25602681 PMC4385712

[B3] Afonina E. Neumann M. Pavlakis G. N. (1997). Preferential Binding of Poly(A)-Binding Protein 1 to an Inhibitory RNA Element in the Human Immunodeficiency Virus Type 1 Gag mRNA. J. Biol. Chem. 272 (4), 2307–2311. doi: 10.1074/jbc.272.4.2307 8999938

[B4] Ahlenstiel C. L. Symonds G. Kent S. J. Kelleher A. D. (2020). Block and Lock HIV Cure Strategies to Control the Latent Reservoir. Front. Cell Infect. Microbiol. 10, 424. doi: 10.3389/fcimb.2020.00424 32923412 PMC7457024

[B5] Ait-Ammar A. Kula A. Darcis G. Verdikt R. De Wit S. Gautier V.. (2019). Current Status of Latency Reversing Agents Facing the Heterogeneity of HIV-1 Cellular and Tissue Reservoirs. Front. Microbiol. 10, 3060. doi: 10.3389/fmicb.2019.03060 32038533 PMC6993040

[B6] Ajamian L. Abel K. Rao S. Vyboh K. Garcia-de-Gracia F. Soto-Rifo R.. (2015). HIV-1 Recruits UPF1 But Excludes UPF2 to Promote Nucleocytoplasmic Export of the Genomic RNA. Biomolecules 5 (4), 2808–2839. doi: 10.3390/biom5042808 26492277 PMC4693258

[B7] Ajamian L. Abrahamyan L. Milev M. Ivanov P. V. Kulozik A. E. Gehring N. H.. (2008). Unexpected Roles for UPF1 in HIV-1 RNA Metabolism and Translation. RNA 14 (5), 914–927. doi: 10.1261/rna.829208 18369187 PMC2327365

[B8] Akiyama H. Miller C. M. Ettinger C. R. Belkina A. C. Snyder-Cappione J. E. Gummuluru S. (2018). HIV-1 Intron-Containing RNA Expression Induces Innate Immune Activation and T Cell Dysfunction. Nat. Commun. 9 (1), 3450. doi: 10.1038/s41467-018-05899-7 30150664 PMC6110775

[B9] Amorim R. Costa S. M. Cavaleiro N. P. da Silva E. E. da Costa L. J. (2014). HIV-1 Transcripts Use IRES-Initiation Under Conditions Where Cap-Dependent Translation is Restricted by Poliovirus 2a Protease. PloS One 9 (2), e88619. doi: 10.1371/journal.pone.0088619 24520405 PMC3919812

[B10] Arrigo S. J. Chen I. S. (1991). Rev is Necessary for Translation But Not Cytoplasmic Accumulation of HIV-1 Vif, Vpr, and Env/Vpu 2 RNAs. Genes Dev. 5 (5), 808–819. doi: 10.1101/gad.5.5.808 1827422

[B11] Askjaer P. Jensen T. H. Nilsson J. Englmeier L. Kjems J. (1998). The Specificity of the CRM1-Rev Nuclear Export Signal Interaction Is Mediated by RanGTP. J. Biol. Chem. 273 (50), 33414–33422. doi: 10.1074/jbc.273.50.33414 9837918

[B12] Bakkour N. Lin Y. L. Maire S. Ayadi L. Mahuteau-Betzer F. Nguyen C. H.. (2007). Small-Molecule Inhibition of HIV pre-mRNA Splicing as a Novel Antiretroviral Therapy to Overcome Drug Resistance. PloS Pathog. 3 (10), 1530–1539. doi: 10.1371/journal.ppat.0030159 17967062 PMC2042022

[B13] Balachandran S. Roberts P. C. Kipperman T. Bhalla K. N. Compans R. W. Archer D. R.. (2000). Alpha/beta Interferons Potentiate Virus-Induced Apoptosis Through Activation of the FADD/Caspase-8 Death Signaling Pathway. J. Virol. 74 (3), 1513–1523. doi: 10.1128/JVI.74.3.1513-1523.2000 10627563 PMC111487

[B14] Balasubramaniam M. Pandhare J. Dash C. (2018). Are microRNAs Important Players in HIV-1 Infection? An Update. Viruses 10 (3), E110. doi: 10.3390/v10030110 29510515 PMC5869503

[B15] Bauren G. Belikov S. Wieslander L. (1998). Transcriptional Termination in the Balbiani Ring 1 Gene is Closely Coupled to 3'-End Formation and Excision of the 3'-Terminal Intron. Genes Dev. 12 (17), 2759–2769. doi: 10.1101/gad.12.17.2759 9732273 PMC317118

[B16] Berg R. K. Melchjorsen J. Rintahaka J. Diget E. Soby S. Horan K. A.. (2012). Genomic HIV RNA Induces Innate Immune Responses Through RIG-I-Dependent Sensing of Secondary-Structured RNA. PloS One 7 (1), e29291. doi: 10.1371/journal.pone.0029291 22235281 PMC3250430

[B17] Berkhout B. Ooms M. Beerens N. Huthoff H. Southern E. Verhoef K. (2002). *In Vitro* Evidence That the Untranslated Leader of the HIV-1 Genome is an RNA Checkpoint That Regulates Multiple Functions Through Conformational Changes. J. Biol. Chem. 277 (22), 19967–19975. doi: 10.1074/jbc.M200950200 11896057

[B18] Besnard E. Hakre S. Kampmann M. Lim H. W. Hosmane N. N. Martin A.. (2016). The mTOR Complex Controls HIV Latency. Cell Host Microbe 20 (6), 785–797. doi: 10.1016/j.chom.2016.11.001 27978436 PMC5354304

[B19] Beyer A. L. Osheim Y. N. (1988). Splice Site Selection, Rate of Splicing, and Alternative Splicing on Nascent Transcripts. Genes Dev. 2 (6), 754–765. doi: 10.1101/gad.2.6.754 3138163

[B20] Black A. C. Luo J. Chun S. Bakker A. Fraser J. K. Rosenblatt J. D. (1996). Specific Binding of Polypyrimidine Tract Binding Protein and hnRNP A1 to HIV-1 CRS Elements. Virus Genes 12 (3), 275–285. doi: 10.1007/BF00284648 8883365

[B21] Bohne J. Wodrich H. Krausslich H. G. (2005). Splicing of Human Immunodeficiency Virus RNA is Position-Dependent Suggesting Sequential Removal of Introns From the 5' End. Nucleic Acids Res. 33 (3), 825–837. doi: 10.1093/nar/gki185 15701754 PMC549389

[B22] Boons E. Vanstreels E. Jacquemyn M. Nogueira T. C. Neggers J. E. Vercruysse T.. (2015). Human Exportin-1 Is a Target for Combined Therapy of HIV and AIDS Related Lymphoma. EBioMedicine 2 (9), 1102–1113. doi: 10.1016/j.ebiom.2015.07.041 26501108 PMC4588406

[B23] Brosseau J. P. Lucier J. F. Lamarche A. A. Shkreta L. Gendron D. Lapointe E.. (2014). Redirecting Splicing With Bifunctional Oligonucleotides. Nucleic Acids Res. 42 (6), e40. doi: 10.1093/nar/gkt1287 24375754 PMC3973305

[B24] Butera S. T. Roberts B. D. Lam L. Hodge T. Folks T. M. (1994). Human Immunodeficiency Virus Type 1 RNA Expression by Four Chronically Infected Cell Lines Indicates Multiple Mechanisms of Latency. J. Virol. 68 (4), 2726–2730. doi: 10.1128/JVI.68.4.2726-2730.1994 7511177 PMC236750

[B25] Campos N. Myburgh R. Garcel A. Vautrin A. Lapasset L. Nadal E. S.. (2015). Long Lasting Control of Viral Rebound With a New Drug ABX464 Targeting Rev - Mediated Viral RNA Biogenesis. Retrovirology 12, 30. doi: 10.1186/s12977-015-0159-3 25889234 PMC4422473

[B26] Caputi M. (2011). “The Regulation of HIV-1 mRNA Biogenesis,” in RNA Processing. Ed. Grabowski P. (London, United Kingdom: IntechOpen).

[B27] Caputi M. Mayeda A. Krainer A. R. Zahler A. M. (1999). hnRNP a/B Proteins are Required for Inhibition of HIV-1 Pre-mRNA Splicing. EMBO J. 18 (14), 4060–4067. doi: 10.1093/emboj/18.14.4060 10406810 PMC1171481

[B28] Caputi M. Zahler A. M. (2002). SR Proteins and hnRNP H Regulate the Splicing of the HIV-1 Tev-Specific Exon 6d. EMBO J. 21 (4), 845–855. doi: 10.1093/emboj/21.4.845 11847131 PMC125874

[B29] Carabet L. A. Leblanc E. Lallous N. Morin H. Ghaidi F. Lee J.. (2019). Computer-Aided Discovery of Small Molecules Targeting the RNA Splicing Activity of hnRNP A1 in Castration-Resistant Prostate Cancer. Molecules 24 (4), 763. doi; 10.3390/molecules24040763 PMC6413181 30791548

[B30] Chatel-Chaix L. Clement J. F. Martel C. Beriault V. Gatignol A. DesGroseillers L.. (2004). Identification of Staufen in the Human Immunodeficiency Virus Type 1 Gag Ribonucleoprotein Complex and a Role in Generating Infectious Viral Particles. Mol. Cell Biol. 24 (7), 2637–2648. doi: 10.1128/MCB.24.7.2637-2648.2004 15024055 PMC371130

[B31] Chawla-Sarkar M. Lindner D. J. Liu Y. F. Williams B. R. Sen G. C. Silverman R. H.. (2003). Apoptosis and Interferons: Role of Interferon-Stimulated Genes as Mediators of Apoptosis. Apoptosis 8 (3), 237–249. doi: 10.1023/a:1023668705040 12766484

[B32] Chen S. Kumar S. Espada C. E. Tirumuru N. Cahill M. P. Hu L.. (2021). N6-Methyladenosine Modification of HIV-1 RNA Suppresses Type-I Interferon Induction in Differentiated Monocytic Cells and Primary Macrophages. PloS Pathog. 17 (3), e1009421. doi: 10.1371/journal.ppat.1009421 33690734 PMC7984636

[B33] Chen M. Li M. Budai M. M. Rice A. P. Kimata J. T. Mohan M.. (2022). Clearance of HIV-1 or SIV Reservoirs by Promotion of Apoptosis and Inhibition of Autophagy: Targeting Intracellular Molecules in Cure-Directed Strategies. J. Leukoc. Biol. 2022, 1–15. doi: 10.1002/JLB.4MR0222-606 PMC9522917 35362118

[B34] Chen J. Umunnakwe C. Sun D. Q. Nikolaitchik O. A. Pathak V. K. Berkhout B.. (2020). Impact of Nuclear Export Pathway on Cytoplasmic HIV-1 RNA Transport Mechanism and Distribution. mBio 11 (6):e01578–20. doi: 10.1128/mBio.01578-20 33172997 PMC7667035

[B35] Cheung P. K. Horhant D. Bandy L. E. Zamiri M. Rabea S. M. Karagiosov S. K.. (2016). A Parallel Synthesis Approach to the Identification of Novel Diheteroarylamide-Based Compounds Blocking HIV Replication: Potential Inhibitors of HIV-1 Pre-mRNA Alternative Splicing. J. Med. Chem. 59 (5), 1869–1879. doi: 10.1021/acs.jmedchem.5b01357 26878150

[B36] Chiang K. Sung T. L. Rice A. P. (2012). Regulation of Cyclin T1 and HIV-1 Replication by microRNAs in Resting CD4+ T Lymphocytes. J. Virol. 86 (6), 3244–3252. doi: 10.1128/JVI.05065-11 22205749 PMC3302325

[B37] Chintala K. Mohareer K. Banerjee S. (2021). Dodging the Host Interferon-Stimulated Gene Mediated Innate Immunity by HIV-1: A Brief Update on Intrinsic Mechanisms and Counter-Mechanisms. Front. Immunol. 12, 716927. doi: 10.3389/fimmu.2021.716927 34394123 PMC8358655

[B38] Chun T. W. Moir S. Fauci A. S. (2015). HIV Reservoirs as Obstacles and Opportunities for an HIV Cure. Nat. Immunol. 16 (6), 584–589. doi: 10.1038/ni.3152 25990814

[B39] Cochrane A. W. Jones K. S. Beidas S. Dillon P. J. Skalka A. M. Rosen C. A. (1991). Identification and Characterization of Intragenic Sequences Which Repress Human Immunodeficiency Virus Structural Gene Expression. J. Virol. 65 (10), 5305–5313. doi: 10.1128/JVI.65.10.5305-5313.1991 1895385 PMC249010

[B40] Cochrane A. W. McNally M. T. Mouland A. J. (2006). The Retrovirus RNA Trafficking Granule: From Birth to Maturity. Retrovirology 3, 18. doi: 10.1186/1742-4690-3-18 16545126 PMC1475878

[B41] Contreras X. Salifou K. Sanchez G. Helsmoortel M. Beyne E. Bluy L.. (2018). Nuclear RNA Surveillance Complexes Silence HIV-1 Transcription. PloS Pathog. 14 (3), e1006950. doi: 10.1371/journal.ppat.1006950 29554134 PMC5875879

[B42] Cordin O. Beggs J. D. (2013). RNA Helicases in Splicing. RNA Biol. 10 (1), 83–95. doi: 10.4161/rna.22547 23229095 PMC3590240

[B43] Courtney D. G. Tsai K. Bogerd H. P. Kennedy E. M. Law B. A. Emery A.. (2019). Epitranscriptomic Addition of M(5)C to HIV-1 Transcripts Regulates Viral Gene Expression. Cell Host Microbe 26 (2), 217–227.e6. doi: 10.1016/j.chom.2019.07.005 31415754 PMC6714563

[B44] Cristinelli S. Angelino P. Janowczyk A. Delorenzi M. Ciuffi A. (2021). HIV Modifies the M6a and M5c Epitranscriptomic Landscape of the Host Cell. Front. Virol. 1 (11). doi: 10.3389/fviro.2021.714475

[B45] Cullen B. R. (2003). Nuclear mRNA Export: Insights From Virology. Trends Biochem. Sci. 28 (8), 419–424. doi: 10.1016/S0968-0004(03)00142-7 12932730

[B46] Daelemans D. Afonina E. Nilsson J. Werner G. Kjems J. De Clercq E.. (2002). A Synthetic HIV-1 Rev Inhibitor Interfering With the CRM1-Mediated Nuclear Export. Proc. Natl. Acad. Sci. U. S. A. 99 (22), 14440–14445. doi: 10.1073/pnas.212285299 12374846 PMC137902

[B47] Daelemans D. Boons E. Vanstreels E. Jacquemyn M. Nogueira T. C. Neggers J. E.. (2015). Selective Inhibitors of Nuclear Export (SINE) Compounds Suppress Both HIV Replication and AIDS Related Lymphoma. Blood 126 (23), 2751. doi: 10.1182/blood.V126.23.2751.2751

[B48] Dahal S. Cheng R. Cheung P. K. Been T. Malty R. Geng M.. (2022). The Thiazole-5-Carboxamide GPS491 Inhibits HIV-1, Adenovirus, and Coronavirus Replication by Altering RNA Processing/Accumulation. Viruses 14 (1), 60. doi: 10.3390/v14010060 PMC8779516 35062264

[B49] Das A. T. Harwig A. Berkhout B. (2011). The HIV-1 Tat Protein has a Versatile Role in Activating Viral Transcription. J. Virol. 85 (18), 9506–9516. doi: 10.1128/JVI.00650-11 21752913 PMC3165771

[B50] De Crignis E. Mahmoudi T. (2017). The Multifaceted Contributions of Chromatin to HIV-1 Integration, Transcription, and Latency. Int. Rev. Cell Mol. Biol. 328, 197–252. doi: 10.1016/bs.ircmb.2016.08.006 28069134

[B51] Deeks S. G. (2012). HIV: Shock and Kill. Nature 487 (7408), 439–440. doi: 10.1038/487439a 22836995

[B52] Deeks S. G. Archin N. Cannon P. Collins S. Jones R. B. de Jong M.. (2021). Research Priorities for an HIV Cure: International AIDS Society Global Scientific Strategy 2021. Nat. Med. 27 (12), 2085–2098. doi: 10.1038/s41591-021-01590-5 34848888

[B53] DeJong E. S. Chang C. E. Gilson M. K. Marino J. P. (2003). Proflavine Acts as a Rev Inhibitor by Targeting the High-Affinity Rev Binding Site of the Rev Responsive Element of HIV-1. Biochemistry 42 (26), 8035–8046. doi: 10.1021/bi034252z 12834355

[B54] Del Corpo O. Goguen R. P. Malard C. M. G. Daher A. Colby-Germinario S. Scarborough R. J.. (2019). A U1i RNA That Enhances HIV-1 RNA Splicing With an Elongated Recognition Domain Is an Optimal Candidate for Combination HIV-1 Gene Therapy. Mol. Ther. Nucleic Acids 18, 815–830. doi: 10.1016/j.omtn.2019.10.011 31734561 PMC6861678

[B55] Dlamini Z. Hull R. (2017). Can the HIV-1 Splicing Machinery be Targeted for Drug Discovery? HIV AIDS (Auckl) 9, 63–75. doi: 10.2147/HIV.S120576 28331370 PMC5354533

[B56] Dufour C. Gantner P. Fromentin R. Chomont N. (2020). The Multifaceted Nature of HIV Latency. J. Clin. Invest 130 (7), 3381–3390. doi: 10.1172/JCI136227 32609095 PMC7324199

[B57] Dugré-Brisson S. Elvira G. Boulay K. Chatel-Chaix L. Mouland A. J. DesGroseillers L. (2005). Interaction of Staufen1 With the 5' End of mRNA Facilitates Translation of These RNAs. Nucleic Acids Res. 33 (15), 4797–4812. doi: 10.1093/nar/gki794 16126845 PMC1193567

[B58] Durand S. Cougot N. Mahuteau-Betzer F. Nguyen C. H. Grierson D. S. Bertrand E.. (2007). Inhibition of Nonsense-Mediated mRNA Decay (NMD) by a New Chemical Molecule Reveals the Dynamic of NMD Factors in P-Bodies. J. Cell Biol. 178 (7), 1145–1160. doi: 10.1083/jcb.200611086 17893241 PMC2064650

[B59] Elliott J. H. Wightman F. Solomon A. Ghneim K. Ahlers J. Cameron M. J.. (2014). Activation of HIV Transcription With Short-Course Vorinostat in HIV-Infected Patients on Suppressive Antiretroviral Therapy. PloS Pathog. 10 (10), e1004473. doi: 10.1371/journal.ppat.1004473 25393648 PMC4231123

[B60] Emery A. Zhou S. Pollom E. Swanstrom R. (2017). Characterizing HIV-1 Splicing by Using Next-Generation Sequencing. J. Virol. 91 (6), e02515–16. doi: 10.1128/JVI.02515-16 28077653 PMC5331825

[B61] Erkelenz S. Hillebrand F. Widera M. Theiss S. Fayyaz A. Degrandi D.. (2015). Balanced Splicing at the Tat-Specific HIV-1 3'ss A3 Is Critical for HIV-1 Replication. Retrovirology 12, 29. doi: 10.1186/s12977-015-0154-8 25889056 PMC4422144

[B62] Fang J. Acheampong E. Dave R. Wang F. Mukhtar M. Pomerantz R. J. (2005). The RNA Helicase DDX1 is Involved in Restricted HIV-1 Rev Function in Human Astrocytes. Virology 336 (2), 299–307. doi: 10.1016/j.virol.2005.03.017 15892970

[B63] Finzi D. Hermankova M. Pierson T. Carruth L. M. Buck C. Chaisson R. E.. (1997). Identification of a Reservoir for HIV-1 in Patients on Highly Active Antiretroviral Therapy. Science 278 (5341), 1295–1300. doi: 10.1126/science.278.5341.1295 9360927

[B64] Fischer M. Wong J. K. Russenberger D. Joos B. Opravil M. Hirschel B.. (2002). Residual Cell-Associated Unspliced HIV-1 RNA in Peripheral Blood of Patients on Potent Antiretroviral Therapy Represents Intracellular Transcripts. Antivir Ther. 7 (2), 91–103. doi: 10.1177/135965350200700203 12212929

[B65] Fong L. E. Sulistijo E. S. Miller-Jensen K. (2017). Systems Analysis of Latent HIV Reversal Reveals Altered Stress Kinase Signaling and Increased Cell Death in Infected T Cells. Sci. Rep. 7 (1), 16179. doi: 10.1038/s41598-017-15532-0 29170390 PMC5701066

[B66] Frohlich A. Rojas-Araya B. Pereira-Montecinos C. Dellarossa A. Toro-Ascuy D. Prades-Perez Y.. (2016). DEAD-Box RNA Helicase DDX3 Connects CRM1-Dependent Nuclear Export and Translation of the HIV-1 Unspliced mRNA Through its N-Terminal Domain. Biochim. Biophys. Acta 1859 (5), 719–730. doi: 10.1016/j.bbagrm.2016.03.009 27012366

[B67] Fukuhara T. Hosoya T. Shimizu S. Sumi K. Oshiro T. Yoshinaka Y.. (2006). Utilization of Host SR Protein Kinases and RNA-Splicing Machinery During Viral Replication. Proc. Natl. Acad. Sci. U. S. A. 103 (30), 11329–11333. doi: 10.1073/pnas.0604616103 16840555 PMC1544086

[B68] Garcia-de-Gracia F. Gaete-Argel A. Riquelme-Barrios S. Pereira-Montecinos C. Rojas-Araya B. Aguilera P.. (2021). CBP80/20-Dependent Translation Initiation Factor (CTIF) Inhibits HIV-1 Gag Synthesis by Targeting the Function of the Viral Protein Rev. RNA Biol. 18 (5), 745–758. doi: 10.1080/15476286.2020.1832375 33103564 PMC8078705

[B69] Garcia-Vidal E. Castellvi M. Pujantell M. Badia R. Jou A. Gomez L.. (2017). Evaluation of the Innate Immune Modulator Acitretin as a Strategy To Clear the HIV Reservoir. Antimicrob. Agents Chemother. 61 (11), e01368–17. doi: 10.1128/AAC.01368-17 28874382 PMC5655051

[B70] Golumbeanu M. Cristinelli S. Rato S. Munoz M. Cavassini M. Beerenwinkel N.. (2018). Single-Cell RNA-Seq Reveals Transcriptional Heterogeneity in Latent and Reactivated HIV-Infected Cells. Cell Rep. 23 (4), 942–950. doi: 10.1016/j.celrep.2018.03.102 29694901

[B71] Gonatopoulos-Pournatzis T. Dunn S. Bounds R. Cowling V. H. (2011). RAM/Fam103a1 Is Required for mRNA Cap Methylation. Mol. Cell. 44 (4), 585–596. doi: 10.1016/j.molcel.2011.08.041 22099306 PMC3235549

[B72] Grau-Exposito J. Luque-Ballesteros L. Navarro J. Curran A. Burgos J. Ribera E.. (2019). Latency Reversal Agents Affect Differently the Latent Reservoir Present in Distinct CD4+ T Subpopulations. PloS Pathog. 15 (8), e1007991. doi: 10.1371/journal.ppat.1007991 31425551 PMC6715238

[B73] Graveley B. R. (2000). Sorting Out the Complexity of SR Protein Functions. RNA 6 (9), 1197–1211. doi: 10.1017/S1355838200000960 10999598 PMC1369994

[B74] Guerrero S. Batisse J. Libre C. Bernacchi S. Marquet R. Paillart J. C. (2015). HIV-1 Replication and the Cellular Eukaryotic Translation Apparatus. Viruses 7 (1), 199–218. doi: 10.3390/v7010199 25606970 PMC4306834

[B75] Heath C. G. Viphakone N. Wilson S. A. (2016). The Role of TREX in Gene Expression and Disease. Biochem. J. 473 (19), 2911–2935. doi: 10.1042/BCJ20160010 27679854 PMC5095910

[B76] Heinson A. I. Woo J. Mukim A. White C. H. Moesker B. Bosque A.. (2021). Micro RNA Targets in HIV Latency: Insights Into Novel Layers of Latency Control. AIDS Res. Hum. Retroviruses 37 (2), 109–121. doi: 10.1089/aid.2020.0150 33045840 PMC7876363

[B77] Henderson B. R. Percipalle P. (1997). Interactions Between HIV Rev and Nuclear Import and Export Factors: The Rev Nuclear Localisation Signal Mediates Specific Binding to Human Importin-Beta. J. Mol. Biol. 274 (5), 693–707. doi: 10.1006/jmbi.1997.1420 9405152

[B78] Henrich T. J. Schreiner C. Cameron C. Hogan L. E. Richardson B. Rutishauser R. L.. (2021). Everolimus, an Mtorc1/2 Inhibitor, in ART-Suppressed Individuals Who Received Solid Organ Transplantation: A Prospective Study. Am. J. Transpl 21 (5), 1765–1779. doi: 10.1111/ajt.16244 PMC9177122 32780519

[B79] Hentze M. W. (1991). Determinants and Regulation of Cytoplasmic mRNA Stability in Eukaryotic Cells. Biochim. Biophys. Acta 1090 (3), 281–292. doi: 10.1016/0167-4781(91)90191-N 1954250

[B80] Hermankova M. Siliciano J. D. Zhou Y. Monie D. Chadwick K. Margolick J. B.. (2003). Analysis of Human Immunodeficiency Virus Type 1 Gene Expression in Latently Infected Resting CD4+ T Lymphocytes In Vivo. J. Virol. 77 (13), 7383–7392. doi: 10.1128/JVI.77.13.7383-7392.2003 12805437 PMC164778

[B81] Ho Y. C. Shan L. Hosmane N. N. Wang J. Laskey S. B. Rosenbloom D. I.. (2013). Replication-Competent Noninduced Proviruses in the Latent Reservoir Increase Barrier to HIV-1 Cure. Cell 155 (3), 540–551. doi: 10.1016/j.cell.2013.09.020 24243014 PMC3896327

[B82] Houzet L. Klase Z. Yeung M. L. Wu A. Le S. Y. Quinones M.. (2012). The Extent of Sequence Complementarity Correlates With the Potency of Cellular miRNA-Mediated Restriction of HIV-1. Nucleic Acids Res. 40 (22), 11684–11696. doi: 10.1093/nar/gks912 23042677 PMC3526334

[B83] Huang Y. Steitz J. A. (2005). SRprises Along a Messenger's Journey. Mol. Cell. 17 (5), 613–615. doi: 10.1016/j.molcel.2005.02.020 15749011

[B84] Huang J. Wang F. Argyris E. Chen K. Liang Z. Tian H.. (2007). Cellular microRNAs Contribute to HIV-1 Latency in Resting Primary CD4+ T Lymphocytes. Nat. Med. 13 (10), 1241–1247. doi: 10.1038/nm1639 17906637

[B85] Huthoff H. Berkhout B. (2001). Two Alternating Structures of the HIV-1 Leader RNA. RNA 7 (1), 143–157. doi: 10.1017/S1355838201001881 11214176 PMC1370064

[B86] Jablonski J. A. Amelio A. L. Giacca M. Caputi M. (2010). The Transcriptional Transactivator Tat Selectively Regulates Viral Splicing. Nucleic Acids Res. 38 (4), 1249–1260. doi: 10.1093/nar/gkp1105 19966273 PMC2831323

[B87] Jablonski J. A. Caputi M. (2009). Role of Cellular RNA Processing Factors in Human Immunodeficiency Virus Type 1 mRNA Metabolism, Replication, and Infectivity. J. Virol. 83 (2), 981–992. doi: 10.1128/JVI.01801-08 19004959 PMC2612387

[B88] Jackson P. E. Tebit D. M. Rekosh D. Hammarskjold M. L. (2016). Rev-RRE Functional Activity Differs Substantially Among Primary HIV-1 Isolates. AIDS Res. Hum. Retroviruses 32 (9), 923–934. doi: 10.1089/aid.2016.0047 27147495 PMC5028908

[B89] Janssen H. L. Reesink H. W. Lawitz E. J. Zeuzem S. Rodriguez-Torres M. Patel K.. (2013). Treatment of HCV Infection by Targeting microRNA. N. Engl. J. Med. 368 (18), 1685–1694. doi: 10.1056/NEJMoa1209026 23534542

[B90] Jiang X. Liu B. Nie Z. Duan L. Xiong Q. Jin Z.. (2021). The Role of M6a Modification in the Biological Functions and Diseases. Signal Transduct Target Ther. 6 (1), 74. doi: 10.1038/s41392-020-00450-x 33611339 PMC7897327

[B91] Jin S. Liao Q. Chen J. Zhang L. He Q. Zhu H.. (2018). TSC1 and DEPDC5 Regulate HIV-1 Latency Through the mTOR Signaling Pathway. Emerg. Microbes Infect. 7 (1), 138. doi: 10.1038/s41426-018-0139-5 30087333 PMC6081400

[B92] Kamori D. Ueno T. (2017). HIV-1 Tat and Viral Latency: What We Can Learn From Naturally Occurring Sequence Variations. Front. Microbiol. 8, 80. doi: 10.3389/fmicb.2017.00080 28194140 PMC5276809

[B93] Karn J. Stoltzfus C. M. (2012). Transcriptional and Posttranscriptional Regulation of HIV-1 Gene Expression. Cold Spring Harb. Perspect. Med. 2 (2), a006916. doi: 10.1101/cshperspect.a006916 22355797 PMC3281586

[B94] Kennedy E. M. Bogerd H. P. Kornepati A. V. R. Kang D. Ghoshal D. Marshall J. B.. (2017). Posttranscriptional M(6)A Editing of HIV-1 mRNAs Enhances Viral Gene Expression. Cell Host Microbe 22 (6), 830. doi: 10.1016/j.chom.2017.11.010 29241043 PMC5746179

[B95] Khoury G. Lee M. Y. Ramarathinam S. H. McMahon J. Purcell A. W. Sonza S.. (2021). The RNA-Binding Proteins SRP14 and HMGB3 Control HIV-1 Tat mRNA Processing and Translation During HIV-1 Latency. Front. Genet. 12, 680725. doi: 10.3389/fgene.2021.680725 34194479 PMC8236859

[B96] Khoury G. Mota T. M. Li S. Tumpach C. Lee M. Y. Jacobson J.. (2018). HIV Latency Reversing Agents Act Through Tat Post Translational Modifications. Retrovirology 15 (1), 36. doi: 10.1186/s12977-018-0421-6 29751762 PMC5948896

[B97] Kilchert C. Wittmann S. Vasiljeva L. (2016). The Regulation and Functions of the Nuclear RNA Exosome Complex. Nat. Rev. Mol. Cell Biol. 17 (4), 227–239. doi: 10.1038/nrm.2015.15 26726035

[B98] Kim Y. Anderson J. L. Lewin S. R. (2018). Getting the "Kill" Into "Shock and Kill": Strategies to Eliminate Latent HIV. Cell Host Microbe 23 (1), 14–26. doi: 10.1016/j.chom.2017.12.004 29324227 PMC5990418

[B99] Kjems J. Frankel A. D. Sharp P. A. (1991). Specific Regulation of mRNA Splicing *In Vitro* by a Peptide From HIV-1 Rev. Cell 67 (1), 169–178. doi: 10.1016/0092-8674(91)90580-R 1913815

[B100] Kjems J. Sharp P. A. (1993). The Basic Domain of Rev From Human Immunodeficiency Virus Type 1 Specifically Blocks the Entry of U4/U6.U5 Small Nuclear Ribonucleoprotein in Spliceosome Assembly. J. Virol. 67 (8), 4769–4776. doi: 10.1128/JVI.67.8.4769-4776.1993 8331728 PMC237863

[B101] Knoener R. A. Becker J. T. Scalf M. Sherer N. M. Smith L. M. (2017). Elucidating the *In Vivo* Interactome of HIV-1 RNA by Hybridization Capture and Mass Spectrometry. Sci. Rep. 7 (1), 16965. doi: 10.1038/s41598-017-16793-5 29208937 PMC5717263

[B102] Knoener R. Evans E. 3rd Becker J. T. Scalf M. Benner B. Sherer N. M.. (2021). Identification of Host Proteins Differentially Associated With HIV-1 RNA Splice Variants. Elife 10, e62470. doi: 10.7554/eLife.62470.sa2 33629952 PMC7906601

[B103] Kohler A. Hurt E. (2007). Exporting RNA From the Nucleus to the Cytoplasm. Nat. Rev. Mol. Cell Biol. 8 (10), 761–773. doi: 10.1038/nrm2255 17786152

[B104] Kong W. Rivera-Serrano E. E. Neidleman J. A. Zhu J. (2019). HIV-1 Replication Benefits From the RNA Epitranscriptomic Code. J. Mol. Biol. 431 (24), 5032–5038. doi: 10.1016/j.jmb.2019.09.021 31626810 PMC6953616

[B105] König R. Zhou Y. Elleder D. Diamond T. L. Bonamy G. M. Irelan J. T.. (2008). Global Analysis of Host-Pathogen Interactions That Regulate Early-Stage HIV-1 Replication. Cell 135 (1), 49–60. doi: 10.1016/j.cell.2008.07.032 18854154 PMC2628946

[B106] Kreider E. F. Bar K. J. (2022). HIV-1 Reservoir Persistence and Decay: Implications for Cure Strategies. Curr. HIV/AIDS Rep 19(3), 194–206. doi: 10.1007/s11904-022-00604-2 35404007 PMC10443186

[B107] Kula A. Gharu L. Marcello A. (2013). HIV-1 pre-mRNA Commitment to Rev Mediated Export Through PSF and Matrin 3. Virology 435 (2), 329–340. doi: 10.1016/j.virol.2012.10.032 23158102

[B108] Kula A. Guerra J. Knezevich A. Kleva D. Myers M. P. Marcello A. (2011). Characterization of the HIV-1 RNA Associated Proteome Identifies Matrin 3 as a Nuclear Cofactor of Rev Function. Retrovirology 8, 60. doi: 10.1186/1742-4690-8-60 21771346 PMC3160904

[B109] Kyei G. B. Meng S. Ramani R. Niu A. Lagisetti C. Webb T. R.. (2018). Splicing Factor 3b Subunit 1 Interacts With HIV Tat and Plays a Role in Viral Transcription and Reactivation From Latency. mBio 9 (6), e01423–18. doi: 10.1128/mBio.01423-18 30401776 PMC6222122

[B110] Lassen K. G. Bailey J. R. Siliciano R. F. (2004). Analysis of Human Immunodeficiency Virus Type 1 Transcriptional Elongation in Resting CD4+ T Cells In Vivo. J. Virol. 78 (17), 9105–9114. doi: 10.1128/JVI.78.17.9105-9114.2004 15308706 PMC506937

[B111] Lassen K. G. Ramyar K. X. Bailey J. R. Zhou Y. Siliciano R. F. (2006). Nuclear Retention of Multiply Spliced HIV-1 RNA in Resting CD4+ T Cells. PloS Pathog. 2 (7), e68. doi: 10.1371/journal.ppat.0020068 16839202 PMC1487174

[B112] Lewin S. R. Vesanen M. Kostrikis L. Hurley A. Duran M. Zhang L.. (1999). Use of Real-Time PCR and Molecular Beacons to Detect Virus Replication in Human Immunodeficiency Virus Type 1-Infected Individuals on Prolonged Effective Antiretroviral Therapy. J. Virol. 73 (7), 6099–6103. doi: 10.1128/JVI.73.7.6099-6103.1999 10364365 PMC112674

[B113] Lichinchi G. Gao S. Saletore Y. Gonzalez G. M. Bansal V. Wang Y.. (2016). Dynamics of the Human and Viral M(6)A RNA Methylomes During HIV-1 Infection of T Cells. Nat. Microbiol. 1, 16011. doi: 10.1038/nmicrobiol.2016.11 27572442 PMC6053355

[B114] Li P. Kaiser P. Lampiris H. W. Kim P. Yukl S. A. Havlir D. V.. (2016). Stimulating the RIG-I Pathway to Kill Cells in the Latent HIV Reservoir Following Viral Reactivation. Nat. Med. 22 (7), 807–811. doi: 10.1038/nm.4124 27294875 PMC5004598

[B115] Li M. Kao E. Gao X. Sandig H. Limmer K. Pavon-Eternod M.. (2012). Codon-Usage-Based Inhibition of HIV Protein Synthesis by Human Schlafen 11. Nature 491 (7422), 125–128. doi: 10.1038/nature11433 23000900 PMC3705913

[B116] Li X. D. Moore B. Cloyd M. W. (1996). Gradual Shutdown of Virus Production Resulting in Latency Is the Norm During the Chronic Phase of Human Immunodeficiency Virus Replication and Differential Rates and Mechanisms of Shutdown Are Determined by Viral Sequences. Virology 225 (1), 196–212. doi: 10.1006/viro.1996.0588 8918547

[B117] Lin X. Irwin D. Kanazawa S. Huang L. Romeo J. Yen T. S.. (2003). Transcriptional Profiles of Latent Human Immunodeficiency Virus in Infected Individuals: Effects of Tat on the Host and Reservoir. J. Virol. 77 (15), 8227–8236. doi: 10.1128/JVI.77.15.8227-8236.2003 12857891 PMC165222

[B118] Liu G. Lu Y. Thulasi Raman S. N. Xu F. Wu Q. Li Z.. (2018). Nuclear-Resident RIG-I Senses Viral Replication Inducing Antiviral Immunity. Nat. Commun. 9 (1), 3199. doi: 10.1038/s41467-018-05745-w 30097581 PMC6086882

[B119] Liu R. Yeh Y. J. Varabyou A. Collora J. A. Sherrill-Mix S. Talbot C. C. Jr.. (2020). Single-Cell Transcriptional Landscapes Reveal HIV-1-Driven Aberrant Host Gene Transcription as a Potential Therapeutic Target. Sci. Transl. Med. 12 (543), eaaz0802. doi: 10.1126/scitranslmed.aaz0802 32404504 PMC7453882

[B120] Lopez-Huertas M. R. Morin M. Madrid-Elena N. Gutierrez C. Jimenez-Tormo L. Santoyo J.. (2019). Selective miRNA Modulation Fails to Activate HIV Replication in *In Vitro* Latency Models. Mol. Ther. Nucleic Acids 17, 323–336. doi: 10.1016/j.omtn.2019.06.006 31288207 PMC6614709

[B121] Luo Y. Jacobs E. Y. Greco T. M. Mohammed K. D. Tong T. Keegan S.. (2016). HIV-Host Interactome Revealed Directly From Infected Cells. Nat. Microbiol. 1 (7), 16068. doi: 10.1038/nmicrobiol.2016.68 27375898 PMC4928716

[B122] Lu W. Tirumuru N. St Gelais C. Koneru P. C. Liu C. Kvaratskhelia M.. (2018). N(6)-Methyladenosine-Binding Proteins Suppress HIV-1 Infectivity and Viral Production. J. Biol. Chem. 293 (34), 12992–13005. doi: 10.1074/jbc.RA118.004215 29976753 PMC6109920

[B123] Maldarelli F. Martin M. A. Strebel K. (1991). Identification of Posttranscriptionally Active Inhibitory Sequences in Human Immunodeficiency Virus Type 1 RNA: Novel Level of Gene Regulation. J. Virol. 65 (11), 5732–5743. doi: 10.1128/jvi.65.11.5732-5743.1991 1656066 PMC250233

[B124] Malim M. H. Cullen B. R. (1991). HIV-1 Structural Gene Expression Requires the Binding of Multiple Rev Monomers to the Viral RRE: Implications for HIV-1 Latency. Cell 65 (2), 241–248. doi: 10.1016/0092-8674(91)90158-U 2015625

[B125] Malim M. H. Hauber J. Le S. Y. Maizel J. V. Cullen B. R. (1989). The HIV-1 Rev Trans-Activator Acts Through a Structured Target Sequence to Activate Nuclear Export of Unspliced Viral mRNA. Nature 338 (6212), 254–257. doi: 10.1038/338254a0 2784194

[B126] Mancebo H. S. Lee G. Flygare J. Tomassini J. Luu P. Zhu Y.. (1997). P-TEFb Kinase Is Required for HIV Tat Transcriptional Activation *In Vivo* and In Vitro. Genes Dev. 11 (20), 2633–2644. doi: 10.1101/gad.11.20.2633 9334326 PMC316604

[B127] Manley J. L. Krainer A. R. (2010). A Rational Nomenclature for Serine/Arginine-Rich Protein Splicing Factors (SR Proteins). Genes Dev. 24 (11), 1073–1074. doi: 10.1101/gad.1934910 20516191 PMC2878644

[B128] Mantri C. K. Pandhare Dash J. Mantri J. V. Dash C. C. (2012). Cocaine Enhances HIV-1 Replication in CD4+ T Cells by Down-Regulating MiR-125b. PloS One 7 (12), e51387. doi: 10.1371/journal.pone.0051387 23251514 PMC3520918

[B129] McCauley S. M. Kim K. Nowosielska A. Dauphin A. Yurkovetskiy L. Diehl W. E.. (2018). Intron-Containing RNA From the HIV-1 Provirus Activates Type I Interferon and Inflammatory Cytokines. Nat. Commun. 9 (1), 5305. doi: 10.1038/s41467-018-07753-2 30546110 PMC6294009

[B130] Medina-Trillo C. Sedgwick D. M. Herrera L. Beltran M. Moreno A. Barrio P.. (2020). Nucleic Acid Recognition and Antiviral Activity of 1,4-Substituted Terphenyl Compounds Mimicking All Faces of the HIV-1 Rev Protein Positively-Charged Alpha-Helix. Sci. Rep. 10 (1), 7190. 10.1038/s41598-020-64120-2 32346097 PMC7188855

[B131] Meyer K. D. Jaffrey S. R. (2014). The Dynamic Epitranscriptome: N6-Methyladenosine and Gene Expression Control. Nat. Rev. Mol. Cell Biol. 15 (5), 313–326. doi: 10.1038/nrm3785 24713629 PMC4393108

[B132] Michael N. L. Morrow P. Mosca J. Vahey M. Burke D. S. Redfield R. R. (1991). Induction of Human Immunodeficiency Virus Type 1 Expression in Chronically Infected Cells Is Associated Primarily With a Shift in RNA Splicing Patterns. J. Virol. 65 (12), 7084. doi: 10.1128/JVI.65.12.7084-.1991 PMC250840 1942260

[B133] Mikaelian I. Krieg M. Gait M. J. Karn J. (1996). Interactions of INS (CRS) Elements and the Splicing Machinery Regulate the Production of Rev-Responsive mRNAs. J. Mol. Biol. 257 (2), 246–264. doi: 10.1006/jmbi.1996.0160 8609621

[B134] Modem S. Badri K. R. Holland T. C. Reddy T. R. (2005). Sam68 Is Absolutely Required for Rev Function and HIV-1 Production. Nucleic Acids Res. 33 (3), 873–879. doi: 10.1093/nar/gki231 15701759 PMC549398

[B135] Modem S. Reddy T. R. (2008). An Anti-Apoptotic Protein, Hax-1, Inhibits the HIV-1 Rev Function by Altering Its Sub-Cellular Localization. J. Cell Physiol. 214 (1), 14–19. doi: 10.1002/jcp.21305 17929250

[B136] Monette A. Pante N. Mouland A. J. (2011). HIV-1 Remodels the Nuclear Pore Complex. J. Cell Biol. 193 (4), 619–631. doi: 10.1083/jcb.201008064 21576391 PMC3166874

[B137] Monette A. Valiente-Echeverria F. Rivero M. Cohen E. A. Lopez-Lastra M. Mouland A. J. (2013). Dual Mechanisms of Translation Initiation of the Full-Length HIV-1 mRNA Contribute to Gag Synthesis. PloS One 8 (7), e68108. doi: 10.1371/journal.pone.0068108 23861855 PMC3702555

[B138] Moore M. J. Proudfoot N. J. (2009). Pre-mRNA Processing Reaches Back to Transcription and Ahead to Translation. Cell 136 (4), 688–700. doi: 10.1016/j.cell.2009.02.001 19239889

[B139] Moore M. J. Sharp P. A. (1993). Evidence for Two Active Sites in the Spliceosome Provided by Stereochemistry of pre-mRNA Splicing. Nature 365 (6444), 364–368. 8397340 10.1038/365364a0

[B140] Moron-Lopez S. Bernal S. Wong J. K. Martinez-Picado J. Yukl S. A. (2021). ABX464 Decreases the Total HIV Reservoir and HIV Transcription Initiation in CD4 + T Cells From HIV-Infected ART-Suppressed Individuals. Clin. Infect. Dis 24(7), e25738. doi: 10.1038/365364a0 PMC9187309 34436569

[B141] Moron-Lopez S. Kim P. Sogaard O. S. Tolstrup M. Wong J. K. Yukl S. A. (2019). Characterization of the HIV-1 Transcription Profile After Romidepsin Administration in ART-Suppressed Individuals. AIDS 33 (3), 425–431. doi: 10.1097/QAD.0000000000002083 30531314 PMC6374101

[B142] Moron-Lopez S. Telwatte S. Sarabia I. Battivelli E. Montano M. Macedo A. B.. (2020). Human Splice Factors Contribute to Latent HIV Infection in Primary Cell Models and Blood CD4+ T Cells From ART-Treated Individuals. PloS Pathog. 16 (11), e1009060. doi: 10.1371/journal.ppat.1009060 33253324 PMC7728277

[B143] Mueller N. Pasternak A. O. Klaver B. Cornelissen M. Berkhout B. Das A. T. (2018). The HIV-1 Tat Protein Enhances Splicing at the Major Splice Donor Site. J. Virol. 92 (14), e01855–17. doi: 10.1128/JVI.01855-17 29743356 PMC6026763

[B144] Nandagopal N. Roux P. P. (2015). Regulation of Global and Specific mRNA Translation by the mTOR Signaling Pathway. Translation (Austin) 3 (1), e983402. doi: 10.4161/21690731.2014.983402 26779414 PMC4682803

[B145] Ne E. Crespo R. Izquierdo-Lara R. Rao S. Koçer S. Górska A.. (2022). Catchet-MS Identifies IKZF1-Targeting Thalidomide Analogues as Novel HIV-1 Latency Reversal Agents. Nucleic Acids Res. doi: 10.1093/nar/gkac407 PMC9177988 35640596

[B146] Ne E. Palstra R. J. Mahmoudi T. (2018). Transcription: Insights From the HIV-1 Promoter. Int. Rev. Cell Mol. Biol. 335, 191–243. doi: 10.1016/bs.ircmb.2017.07.011 29305013

[B147] Neupane K. Wahab A. Masood A. Faraz T. Bahram S. Ehsan H.. (2021). Profile and Management of Toxicity of Selinexor and Belantamab Mafodotin for the Treatment of Triple Class Refractory Multiple Myeloma. J. Blood Med. 12, 529–550. doi: 10.2147/JBM.S317966 34234609 PMC8257048

[B148] Neville M. Stutz F. Lee L. Davis L. I. Rosbash M. (1997). The Importin-Beta Family Member Crm1p Bridges the Interaction Between Rev and the Nuclear Pore Complex During Nuclear Export. Curr. Biol. 7 (10), 767–775. doi: 10.1016/S0960-9822(06)00335-6 9368759

[B149] Nishizono N. Nair V. (2000). Synthesis of Biomimetic Analogs of Neomycin B: Potential Inhibitors of the HIV RNA Rev Response Element. Nucleosides Nucleotides Nucleic Acids 19 (1-2), 283–295. doi: 10.1080/15257770008033010 10772716

[B150] Nojima T. Gomes T. Grosso A. R. F. Kimura H. Dye M. J. Dhir S.. (2015). Mammalian NET-Seq Reveals Genome-Wide Nascent Transcription Coupled to RNA Processing. Cell 161 (3), 526–540. doi: 10.1016/j.cell.2015.03.027 25910207 PMC4410947

[B151] Norton N. J. Mok H. P. Sharif F. Hirst J. C. Lever A. M. L. (2019). HIV Silencing and Inducibility Are Heterogeneous and Are Affected by Factors Intrinsic to the Virus. mBio 10 (3), e00188–19. doi: 10.1128/mBio.00188-19 31239371 PMC6593397

[B152] Ocwieja K. E. Sherrill-Mix S. Mukherjee R. Custers-Allen R. David P. Brown M.. (2012). Dynamic Regulation of HIV-1 mRNA Populations Analyzed by Single-Molecule Enrichment and Long-Read Sequencing. Nucleic Acids Res. 40 (20), 10345–10355. doi: 10.1093/nar/gks753 22923523 PMC3488221

[B153] Ohe K. Hagiwara M. (2015). Modulation of Alternative Splicing With Chemical Compounds in New Therapeutics for Human Diseases. ACS Chem. Biol. 10 (4), 914–924. doi: 10.1021/cb500697f 25560473

[B154] Ohlmann T. Mengardi C. Lopez-Lastra M. (2014). Translation Initiation of the HIV-1 mRNA. Translation (Austin) 2 (2), e960242. doi: 10.4161/2169074X.2014.960242 26779410 PMC4696476

[B155] Olsen H. S. Cochrane A. W. Rosen C. (1992). Interaction of Cellular Factors With Intragenic Cis-Acting Repressive Sequences Within the HIV Genome. Virology 191 (2), 709–715. doi: 10.1016/0042-6822(92)90246-L 1448921

[B156] Omoto S. Ito M. Tsutsumi Y. Ichikawa Y. Okuyama H. Brisibe E. A.. (2004). HIV-1 Nef Suppression by Virally Encoded microRNA. Retrovirology 1, 44. doi: 10.1186/1742-4690-1-44 15601474 PMC544868

[B157] Ooms M. Huthoff H. Russell R. Liang C. Berkhout B. (2004). A Riboswitch Regulates RNA Dimerization and Packaging in Human Immunodeficiency Virus Type 1 Virions. J. Virol. 78 (19), 10814–10819. doi: 10.1128/JVI.78.19.10814-10819.2004 15367648 PMC516375

[B158] Organization WHO (2022) HIV/AIDS: World Health Organization. Available at: https://www.who.int/news-room/fact-sheets/detail/hiv-aids.

[B159] Ouellet D. L. Vigneault-Edwards J. Letourneau K. Gobeil L. A. Plante I. Burnett J. C.. (2013). Regulation of Host Gene Expression by HIV-1 TAR microRNAs. Retrovirology 10, 86. doi: 10.1186/1742-4690-10-86 23938024 PMC3751525

[B160] Pasternak A. O. Berkhout B. (2018). What do We Measure When We Measure Cell-Associated HIV RNA. Retrovirology 15 (1), 13. doi: 10.1186/s12977-018-0397-2 29378657 PMC5789533

[B161] Pasternak A. O. Berkhout B. (2021). The Splice of Life: Does RNA Processing Have a Role in HIV-1 Persistence? Viruses 13 (9), 1751. doi: 10.3390/v13091751 34578332 PMC8471011

[B162] Patel P. Ansari M. Y. Bapat S. Thakar M. Gangakhedkar R. Jameel S. (2014). The microRNA miR-29a is Associated With Human Immunodeficiency Virus Latency. Retrovirology 11, 108. doi: 10.1186/s12977-014-0108-6 25486977 PMC4269869

[B163] Perales C. Carrasco L. Gonzalez M. E. (2005). Regulation of HIV-1 Env mRNA Translation by Rev Protein. Biochim. Biophys. Acta 1743 (1-2), 169–175. doi: 10.1016/j.bbamcr.2004.09.030 15777852

[B164] Pomerantz R. J. Seshamma T. Trono D. (1992). Efficient Replication of Human Immunodeficiency Virus Type 1 Requires a Threshold Level of Rev: Potential Implications for Latency. J. Virol. 66 (3), 1809–1813. doi: 10.1128/jvi.66.3.1809-1813.1992 1738210 PMC240948

[B165] Pomerantz R. J. Trono D. Feinberg M. B. Baltimore D. (1990). Cells Nonproductively Infected With HIV-1 Exhibit an Aberrant Pattern of Viral RNA Expression: A Molecular Model for Latency. Cell 61 (7), 1271–1276. doi: 10.1016/0092-8674(90)90691-7 2364429

[B166] Popp M. W. Maquat L. E. (2015). Attenuation of Nonsense-Mediated mRNA Decay Facilitates the Response to Chemotherapeutics. Nat. Commun. 6, 6632. doi: 10.1038/ncomms7632 25808464 PMC4375787

[B167] Prado S. Beltran M. Coiras M. Bedoya L. M. Alcami J. Gallego J. (2016). Bioavailable Inhibitors of HIV-1 RNA Biogenesis Identified Through a Rev-Based Screen. Biochem. Pharmacol. 107, 14–28. doi: 10.1016/j.bcp.2016.02.007 26896646

[B168] Prado S. Beltran M. Moreno A. Bedoya L. M. Alcami J. Gallego J. (2018). A Small-Molecule Inhibitor of HIV-1 Rev Function Detected by a Diversity Screen Based on RRE-Rev Interference. Biochem. Pharmacol. 156, 68–77. doi: 10.1016/j.bcp.2018.07.040 30071201

[B169] Purcell D. F. Martin M. A. (1993). Alternative Splicing of Human Immunodeficiency Virus Type 1 mRNA Modulates Viral Protein Expression, Replication, and Infectivity. J. Virol. 67 (11), 6365–6378. doi: 10.1128/jvi.67.11.6365-6378.1993 8411338 PMC238071

[B170] Rao S. Amorim R. Niu M. Breton Y. Tremblay M. J. Mouland A. J. (2019). Host mRNA Decay Proteins Influence HIV-1 Replication and Viral Gene Expression in Primary Monocyte-Derived Macrophages. Retrovirology 16 (1), 3. doi: 10.1186/s12977-019-0465-2 30732620 PMC6367771

[B171] Rao S. Amorim R. Niu M. Temzi A. Mouland A. J. (2018). The RNA Surveillance Proteins UPF1, UPF2 and SMG6 Affect HIV-1 Reactivation at a Post-Transcriptional Level. Retrovirology 15 (1), 42. doi: 10.1186/s12977-018-0425-2 29954456 PMC6022449

[B172] Rao S. Hassine S. Monette A. Amorim R. DesGroseillers L. Mouland A. J. (2019). HIV-1 Requires Staufen1 to Dissociate Stress Granules and to Produce Infectious Viral Particles. Rna 25 (6), 727–736. doi: 10.1261/rna.069351.118 30902835 PMC6521601

[B173] Rao S. Lungu C. Crespo R. Steijaert T. H. Gorska A. Palstra R. J.. (2021). Selective Cell Death in HIV-1-Infected Cells by DDX3 Inhibitors Leads to Depletion of the Inducible Reservoir. Nat. Commun. 12 (1), 2475. doi: 10.1038/s41467-021-22608-z 33931637 PMC8087668

[B174] Rasmussen E. B. Lis J. T. (1993). *In Vivo* Transcriptional Pausing and Cap Formation on Three Drosophila Heat Shock Genes. Proc. Natl. Acad. Sci. U. S. A. 90 (17), 7923–7927. doi: 10.1073/pnas.90.17.7923 8367444 PMC47259

[B175] Rausch J. W. Le Grice S. F. (2015). HIV Rev Assembly on the Rev Response Element (RRE): A Structural Perspective. Viruses 7 (6), 3053–3075. doi: 10.3390/v7062760 26075509 PMC4488727

[B176] Riquelme-Barrios S. Pereira-Montecinos C. Valiente-Echeverría F. Soto-Rifo R. (2018). Emerging Roles of N(6)-Methyladenosine on HIV-1 RNA Metabolism and Viral Replication. Front. Microbiol. 9, 576. doi: 10.3389/fmicb.2018.00576 29643844 PMC5882793

[B177] Röling M. Mollapour Sisakht M. Ne E. Moulos P. Crespo R. Stoszko M.. (2021). A Two-Color Haploid Genetic Screen Identifies Novel Host Factors Involved in HIV-1 Latency. mBio 12 (6), e0298021. doi: 10.1128/mBio.02980-21 34872356 PMC8649776

[B178] Routy J. P. Tremblay C. L. Angel J. B. Trottier B. Rouleau D. Baril J. G.. (2012). Valproic Acid in Association With Highly Active Antiretroviral Therapy for Reducing Systemic HIV-1 Reservoirs: Results From a Multicentre Randomized Clinical Study. HIV Med. 13 (5), 291–296. doi: 10.1111/j.1468-1293.2011.00975.x 22276680

[B179] Roy S. Delling U. Chen C. H. Rosen C. A. Sonenberg N. (1990). A Bulge Structure in HIV-1 TAR RNA is Required for Tat Binding and Tat-Mediated Trans-Activation. Genes Dev. 4 (8), 1365–1373. doi: 10.1101/gad.4.8.1365 2227414

[B180] Ruelas D. S. Chan J. K. Oh E. Heidersbach A. J. Hebbeler A. M. Chavez L.. (2015). MicroRNA-155 Reinforces HIV Latency. J. Biol. Chem. 290 (22), 13736–13748. doi: 10.1074/jbc.M115.641837 25873391 PMC4447952

[B181] Ruhl M. Himmelspach M. Bahr G. M. Hammerschmid F. Jaksche H. Wolff B.. (1993). Eukaryotic Initiation Factor 5A is a Cellular Target of the Human Immunodeficiency Virus Type 1 Rev Activation Domain Mediating Trans-Activation. J. Cell Biol. 123 (6 Pt 1), 1309–1320. doi: 10.1083/jcb.123.6.1309 8253832 PMC2290910

[B182] Sadri Nahand J. Bokharaei-Salim F. Karimzadeh M. Moghoofei M. Karampoor S. Mirzaei H. R.. (2020). MicroRNAs and Exosomes: Key Players in HIV Pathogenesis. HIV Med. 21 (4), 246–278. doi: 10.1111/hiv.12822 31756034 PMC7069804

[B183] Saliou J. M. Bourgeois C. F. Ayadi-Ben Mena L. Ropers D. Jacquenet S. Marchand V.. (2009). Role of RNA Structure and Protein Factors in the Control of HIV-1 Splicing. Front. Biosci. (Landmark Ed) 14, 2714–2729. doi: 10.2741/3408 19273230

[B184] Sannier G. Dube M. Dufour C. Richard C. Brassard N. Delgado G. G.. (2021). Combined Single-Cell Transcriptional, Translational, and Genomic Profiling Reveals HIV-1 Reservoir Diversity. Cell Rep. 36 (9), 109643. doi: 10.1016/j.celrep.2021.109643 34469719

[B185] Sarracino A. Gharu L. Kula A. Pasternak A. O. Avettand-Fenoel V. Rouzioux C.. (2018). Posttranscriptional Regulation of HIV-1 Gene Expression During Replication and Reactivation From Latency by Nuclear Matrix Protein Matr3. mBio 9 (6), e02158-18. doi: 10.1128/mBio.02158-18 PMC6234869 30425153

[B186] Scarborough R. J. Gatignol A. (2017). RNA Interference Therapies for an HIV-1 Functional Cure. Viruses 10 (1) 8. doi: 10.3390/v10010008 PMC5795421 29280961

[B187] Schröder H. C. Ushijima H. Bek A. Merz H. Pfeifer K. Müller W. E. G. (1993). Inhibition of Formation of Rev-RRE Complex by Pyronin Y. Antiviral Chem. Chemother 4 (2), 103–111. doi: 10.1177/095632029300400205

[B188] Schwartz S. Campbell M. Nasioulas G. Harrison J. Felber B. K. Pavlakis G. N. (1992). Mutational Inactivation of an Inhibitory Sequence in Human Immunodeficiency Virus Type 1 Results in Rev-Independent Gag Expression. J. Virol. 66 (12), 7176–7182. doi: 10.1128/jvi.66.12.7176-7182.1992 1433510 PMC240411

[B189] Schwartz S. Felber B. K. Pavlakis G. N. (1992). Distinct RNA Sequences in the Gag Region of Human Immunodeficiency Virus Type 1 Decrease RNA Stability and Inhibit Expression in the Absence of Rev Protein. J. Virol. 66 (1), 150–159. doi: 10.1128/jvi.66.1.150-159.1992 1727477 PMC238270

[B190] Sedore S. C. Byers S. A. Biglione S. Price J. P. Maury W. J. Price D. H. (2007). Manipulation of P-TEFb Control Machinery by HIV: Recruitment of P-TEFb From the Large Form by Tat and Binding of HEXIM1 to TAR. Nucleic Acids Res. 35 (13), 4347–4358. doi: 10.1093/nar/gkm443 17576689 PMC1935001

[B191] Selberg S. Žusinaite E. Herodes K. Seli N. Kankuri E. Merits A.. (2021). HIV Replication Is Increased by RNA Methylation METTL3/METTL14/WTAP Complex Activators. ACS Omega 6 (24), 15957–15963. doi: 10.1021/acsomega.1c01626 34179640 PMC8223420

[B192] Sertznig H. Hillebrand F. Erkelenz S. Schaal H. Widera M. (2018). Behind the Scenes of HIV-1 Replication: Alternative Splicing as the Dependency Factor on the Quiet. Virology 516, 176–188. doi: 10.1016/j.virol.2018.01.011 29407375

[B193] Seshamma T. Bagasra O. Trono D. Baltimore D. Pomerantz R. J. (1992). Blocked Early-Stage Latency in the Peripheral Blood Cells of Certain Individuals Infected With Human Immunodeficiency Virus Type 1. Proc. Natl. Acad. Sci. U. S. A. 89 (22), 10663–10667. doi: 10.1073/pnas.89.22.10663 1279688 PMC50401

[B194] Shkreta L. Blanchette M. Toutant J. Wilhelm E. Bell B. Story B. A.. (2017). Modulation of the Splicing Regulatory Function of SRSF10 by a Novel Compound That Impairs HIV-1 Replication. Nucleic Acids Res. 45 (7), 4051–4067. doi: 10.1093/nar/gkw1223 27928057 PMC5397194

[B195] Siliciano J. D. Siliciano R. F. (2022). *In Vivo* Dynamics of the Latent Reservoir for HIV-1: New Insights and Implications for Cure. Annu. Rev. Pathol. 17, 271–294. doi: 10.1146/annurev-pathol-050520-112001 34736342

[B196] Sithole N. Williams C. A. Abbink T. E. M. Lever A. M. L. (2020). DDX5 Potentiates HIV-1 Transcription as a Co-Factor of Tat. Retrovirology 17 (1), 6. doi: 10.1186/s12977-020-00514-4 32228614 PMC7106839

[B197] Sogaard O. S. Graversen M. E. Leth S. Olesen R. Brinkmann C. R. Nissen S. K.. (2015). The Depsipeptide Romidepsin Reverses HIV-1 Latency In Vivo. PloS Pathog. 11 (9), e1005142. doi: 10.1371/journal.ppat.1005142 26379282 PMC4575032

[B198] Solano-Gonzalez E. Coburn K. M. Yu W. Wilson G. M. Nurmemmedov E. Kesari S.. (2021). Small Molecules Inhibitors of the Heterogeneous Ribonuclear Protein A18 (hnRNP A18): A Regulator of Protein Translation and an Immune Checkpoint. Nucleic Acids Res. 49 (3), 1235–1246. doi: 10.1093/nar/gkaa1254 33398344 PMC7897483

[B199] Soto-Rifo R. Rubilar P. S. Limousin T. de Breyne S. Decimo D. Ohlmann T. (2012). DEAD-Box Protein DDX3 Associates With Eif4f to Promote Translation of Selected mRNAs. EMBO J. 31 (18), 3745–3756. doi: 10.1038/emboj.2012.220 22872150 PMC3442272

[B200] Stewart M. (2019). Structure and Function of the TREX-2 Complex. Subcell Biochem. 93, 461–470. doi: 10.1007/978-3-030-28151-9_15 31939161

[B201] Stock P. G. Barin B. Hatano H. Rogers R. L. Roland M. E. Lee T. H.. (2014). Reduction of HIV Persistence Following Transplantation in HIV-Infected Kidney Transplant Recipients. Am. J. Transpl 14 (5), 1136–1141. doi: 10.1111/ajt.12699 PMC4012326 24698537

[B202] Stoltzfus C. M. (2009). Chapter 1. Regulation of HIV-1 Alternative RNA Splicing and its Role in Virus Replication. Adv. Virus Res. 74, 1–40. doi: 10.1016/S0065-3527(09)74001-1 19698894

[B203] Stoltzfus C. M. Madsen J. M. (2006). Role of Viral Splicing Elements and Cellular RNA Binding Proteins in Regulation of HIV-1 Alternative RNA Splicing. Curr. HIV Res. 4 (1), 43–55. doi: 10.2174/157016206775197655 16454710

[B204] Stoszko M. Ne E. Abner E. Mahmoudi T. (2019). A Broad Drug Arsenal to Attack a Strenuous Latent HIV Reservoir. Curr. Opin. Virol. 38, 37–53. doi: 10.1016/j.coviro.2019.06.001 31323521

[B205] Swaminathan S. Murray D. D. Kelleher A. D. (2012). The Role of microRNAs in HIV-1 Pathogenesis and Therapy. AIDS 26 (11), 1325–1334. doi: 10.1097/QAD.0b013e328352adca 22382145

[B206] Swaminathan G. Navas-Martin S. Martin-Garcia J. (2014). MicroRNAs and HIV-1 Infection: Antiviral Activities and Beyond. J. Mol. Biol. 426 (6), 1178–1197. doi: 10.1016/j.jmb.2013.12.017 24370931

[B207] Telwatte S. Lee S. Somsouk M. Hatano H. Baker C. Kaiser P.. (2018). Gut and Blood Differ in Constitutive Blocks to HIV Transcription, Suggesting Tissue-Specific Differences in the Mechanisms That Govern HIV Latency. PloS Pathog. 14 (11), e1007357. doi: 10.1371/journal.ppat.1007357 30440043 PMC6237391

[B208] Telwatte S. Moron-Lopez S. Aran D. Kim P. Hsieh C. Joshi S.. (2019). Heterogeneity in HIV and Cellular Transcription Profiles in Cell Line Models of Latent and Productive Infection: Implications for HIV Latency. Retrovirology 16 (1), 32. doi: 10.1186/s12977-019-0494-x 31711503 PMC6849327

[B209] Tirumuru N. Zhao B. S. Lu W. Lu Z. He C. Wu L. (2016). N(6)-Methyladenosine of HIV-1 RNA Regulates Viral Infection and HIV-1 Gag Protein Expression. Elife 5, e15528. doi: 10.7554/eLife.15528.021 27371828 PMC4961459

[B210] Toro-Ascuy D. Rojas-Araya B. Valiente-Echeverria F. Soto-Rifo R. (2016). Interactions Between the HIV-1 Unspliced mRNA and Host mRNA Decay Machineries. Viruses 8 (11), 320. doi: 10.3390/v8110320 PMC5127034 27886048

[B211] Triboulet R. Mari B. Lin Y. L. Chable-Bessia C. Bennasser Y. Lebrigand K.. (2007). Suppression of microRNA-Silencing Pathway by HIV-1 During Virus Replication. Science 315 (5818), 1579–1582. doi: 10.1051/medsci/20072367590 17322031

[B212] Truman C. T.-S. Järvelin A. Davis I. Castello A. (2020). HIV Rev-Isited. Open Biol. 10 (12), 200320. doi: 10.1098/rsob.200320 33352061 PMC7776567

[B213] Tsai K. Bogerd H. P. Kennedy E. M. Emery A. Swanstrom R. Cullen B. R. (2021). Epitranscriptomic Addition of M(6)A Regulates HIV-1 RNA Stability and Alternative Splicing. Genes Dev. 35 (13-14), 992–1004. doi: 10.1101/gad.348508.121 34140354 PMC8247604

[B214] Tsai P. Wu G. Baker C. E. Thayer W. O. Spagnuolo R. A. Sanchez R.. (2016). *In Vivo* Analysis of the Effect of Panobinostat on Cell-Associated HIV RNA and DNA Levels and Latent HIV Infection. Retrovirology 13 (1), 36. doi: 10.1186/s12977-016-0268-7 27206407 PMC4875645

[B215] Van Lint C. Bouchat S. Marcello A. (2013). HIV-1 Transcription and Latency: An Update. Retrovirology 10, 67. doi: 10.1186/1742-4690-10-67 23803414 PMC3699421

[B216] Vautrin A. Manchon L. Garcel A. Campos N. Lapasset L. Laaref A. M.. (2019). Both Anti-Inflammatory and Antiviral Properties of Novel Drug Candidate ABX464 are Mediated by Modulation of RNA Splicing. Sci. Rep. 9 (1), 792. doi: 10.1038/s41598-018-37813-y 30692590 PMC6349857

[B217] Verdin E. (1991). DNase I-Hypersensitive Sites are Associated With Both Long Terminal Repeats and With the Intragenic Enhancer of Integrated Human Immunodeficiency Virus Type 1. J. Virol. 65 (12), 6790–6799. doi: 10.1128/jvi.65.12.6790-6799.1991 1942252 PMC250767

[B218] Verdin E. Van Lint C. (1995). Internal Transcriptional Regulatory Elements in HIV-1 and Other Retroviruses. Cell Mol. Biol. (Noisy-le-grand) 41 (3), 365–369. 7580829

[B219] Wagstaff K. M. Sivakumaran H. Heaton S. M. Harrich D. Jans D. A. (2012). Ivermectin is a Specific Inhibitor of Importin Alpha/Beta-Mediated Nuclear Import Able to Inhibit Replication of HIV-1 and Dengue Virus. Biochem. J. 443 (3), 851–856. doi: 10.1042/BJ20120150 22417684 PMC3327999

[B220] Wang P. Qu X. Zhou X. Shen Y. Ji H. Fu Z.. (2015). Two Cellular microRNAs, miR-196b and miR-1290, Contribute to HIV-1 Latency. Virology 486, 228–238. doi: 10.1016/j.virol.2015.09.016 26469550

[B221] Weinberger L. S. Burnett J. C. Toettcher J. E. Arkin A. P. Schaffer D. V. (2005). Stochastic Gene Expression in a Lentiviral Positive-Feedback Loop: HIV-1 Tat Fluctuations Drive Phenotypic Diversity. Cell 122 (2), 169–182. doi: 10.1016/j.cell.2005.06.006 16051143

[B222] Wiegand A. Spindler J. Hong F. F. Shao W. Cyktor J. C. Cillo A. R.. (2017). Single-Cell Analysis of HIV-1 Transcriptional Activity Reveals Expression of Proviruses in Expanded Clones During ART. Proc. Natl. Acad. Sci. U. S. A. 114 (18), E3659–E3E68. doi: 10.1073/pnas.1617961114 28416661 PMC5422779

[B223] Wolff B. Sanglier J. J. Wang Y. (1997). Leptomycin B Is an Inhibitor of Nuclear Export: Inhibition of Nucleo-Cytoplasmic Translocation of the Human Immunodeficiency Virus Type 1 (HIV-1) Rev Protein and Rev-Dependent mRNA. Chem. Biol. 4 (2), 139–147. doi: 10.1016/S1074-5521(97)90257-X 9190288

[B224] Wong R. Balachandran A. Haaland M. Stoilov P. Cochrane A. (2013). HIV-1 RNA Processing as Therapeutic Target: Characterization of Small Molecule Modulators of HIV-1 RNA Processing and Transport. Retrovirology 10, 045. doi: 10.1186/1742-4690-10-S1-O45

[B225] Wong R. W. Balachandran A. Haaland M. Stoilov P. Cochrane A. (2013). Characterization of Novel Inhibitors of HIV-1 Replication That Function *via* Alteration of Viral RNA Processing and Rev Function. Nucleic Acids Res. 41 (20), 9471–9483. doi: 10.1093/nar/gkt727 23945945 PMC3814367

[B226] Wong R. W. Balachandran A. Ostrowski M. A. Cochrane A. (2013). Digoxin Suppresses HIV-1 Replication by Altering Viral RNA Processing. PloS Pathog. 9 (3), e1003241. doi: 10.1371/journal.ppat.1003241 23555254 PMC3610647

[B227] Xiao H. Wyler E. Milek M. Grewe B. Kirchner P. Ekici A.. (2021). CRNKL1 Is a Highly Selective Regulator of Intron-Retaining HIV-1 and Cellular mRNAs. mBio 12 (1), e02525-20. doi: 10.1128/mBio.02525-20 33468685 PMC7845644

[B228] Yamasoba D. Sato K. Ichinose T. Imamura T. Koepke L. Joas S.. (2019). N4BP1 Restricts HIV-1 and its Inactivation by MALT1 Promotes Viral Reactivation. Nat. Microbiol. 4 (9), 1532–1544. doi: 10.1038/s41564-019-0460-3 31133753

[B229] Yang Z. Yang J. Wang J. Lu X. Jin C. Xie T.. (2015). Identify Potential Regulators in HIV-1 Latency by Joint microRNA and mRNA Analysis. Cell Physiol. Biochem. 36 (2), 569–584. doi: 10.1159/000430121 25997625

[B230] Yedavalli V. S. Jeang K. T. (2011a). Rev-Ing Up Post-Transcriptional HIV-1 RNA Expression. RNA Biol. 8 (2), 195–199. doi: 10.4161/rna.8.2.14803 21358275 PMC3127099

[B231] Yedavalli V. S. Jeang K. T. (2011b). Matrin 3 is a Co-Factor for HIV-1 Rev in Regulating Post-Transcriptional Viral Gene Expression. Retrovirology 8, 61. doi: 10.1186/1742-4690-8-61 21771347 PMC3160905

[B232] Yedavalli V. S. Neuveut C. Chi Y. H. Kleiman L. Jeang K. T. (2004). Requirement of DDX3 DEAD Box RNA Helicase for HIV-1 Rev-RRE Export Function. Cell 119 (3), 381–392. doi: 10.1016/j.cell.2004.09.029 15507209

[B233] Yeh Y. J. Jenike K. M. Calvi R. M. Chiarella J. Hoh R. Deeks S. G.. (2020). Filgotinib Suppresses HIV-1-Driven Gene Transcription by Inhibiting HIV-1 Splicing and T Cell Activation. J. Clin. Invest 130 (9), 4969–4984. doi: 10.1172/JCI137371 32573496 PMC7456222

[B234] Yin Y. Zhang S. Luo H. Zhang X. Geng G. Li J.. (2015). Interleukin 7 Up-Regulates CD95 Protein on CD4+ T Cells by Affecting mRNA Alternative Splicing: Priming for a Synergistic Effect on HIV-1 Reservoir Maintenance. J. Biol. Chem. 290 (1), 35–45. doi: 10.1074/jbc.M114.598631 25411246 PMC4281737

[B235] Yukl S. A. Kaiser P. Kim P. Telwatte S. Joshi S. K. Vu M.. (2018). HIV Latency in Isolated Patient CD4(+) T Cells may be Due to Blocks in HIV Transcriptional Elongation, Completion, and Splicing. Sci. Transl. Med. 10 (430), eaap9927. doi: 10.1126/scitranslmed.aap9927 29491188 PMC5959841

[B236] Zerbato J. M. Khoury G. Zhao W. Gartner M. J. Pascoe R. D. Rhodes A.. (2021). Multiply Spliced HIV RNA Is a Predictive Measure of Virus Production Ex Vivo and *In Vivo* Following Reversal of HIV Latency. EBioMedicine 65, 103241. doi: 10.1016/j.ebiom.2021.103241 33647768 PMC7920823

[B237] Zhang H. (2009). Reversal of HIV-1 Latency With anti-microRNA Inhibitors. Int. J. Biochem. Cell Biol. 41 (3), 451–454. doi: 10.1016/j.biocel.2008.07.016 18761423 PMC2723831

[B238] Zhang Q. Kang Y. Wang S. Gonzalez G. M. Li W. Hui H.. (2021). HIV Reprograms Host M(6)Am RNA Methylome by Viral Vpr Protein-Mediated Degradation of PCIF1. Nat. Commun. 12 (1), 5543. doi: 10.1038/s41467-021-25683-4 34545078 PMC8452764

[B239] Zhou X. Luo J. Mills L. Wu S. Pan T. Geng G.. (2013). DDX5 Facilitates HIV-1 Replication as a Cellular Co-Factor of Rev. PloS One 8 (5), e65040. doi: 10.1371/journal.pone.0065040 23741449 PMC3669200

[B240] Zhou Y. Rong L. Zhang J. Aloysius C. Pan Q. Liang C. (2009). Insulin-Like Growth Factor II mRNA Binding Protein 1 Modulates Rev-Dependent Human Immunodeficiency Virus Type 1 RNA Expression. Virology 393 (2), 210–220. doi: 10.1016/j.virol.2009.08.004 19726068

[B241] Zolotukhin A. S. Michalowski D. Bear J. Smulevitch S. V. Traish A. M. Peng R.. (2003). PSF Acts Through the Human Immunodeficiency Virus Type 1 mRNA Instability Elements to Regulate Virus Expression. Mol. Cell Biol. 23 (18), 6618–6630. doi: 10.1128/MCB.23.18.6618-6630.2003 12944487 PMC193712

